# Pixantrone Sensitizes Gram-Negative Pathogens to Rifampin

**DOI:** 10.1128/spectrum.02114-22

**Published:** 2022-11-01

**Authors:** Pengfei She, Zehao Li, Yimin Li, Shasha Liu, Linhui Li, Yifan Yang, Linying Zhou, Yong Wu

**Affiliations:** a Department of Laboratory Medicine, The Third Xiangya Hospital of Central South University, Changsha, Hunan, China; b Department of Laboratory Medicine, The First Hospital of Changsha, Changsha, Hunan, China; Louis Stokes Cleveland VAMC

**Keywords:** drug repurposing, Gram-negative pathogen, pixantrone, proton motive force, reactive oxygen species

## Abstract

The emergence of bacterial drug resistance poses a severe threat to global public health. In particular, antimicrobial-resistant pathogens lead to a high rate of treatment failure and significantly increase mortality. Repurposing FDA-approved compounds to sensitize superbugs to conventional antibiotics provides a promising strategy to alleviate such crises. Pixantrone (PIX) has been approved for treating aggressive B-cell non-Hodgkin’s lymphoma. By high-throughput drug screening, we profiled the synergistic activity between PIX and rifampin (RFP) against Gram-negative extensively drug-resistant isolates by checkerboard assay. Mechanistic studies demonstrated that PIX impacted the flagellum assembly, induced irreversible intracellular reactive oxygen species accumulation and disrupted proton motive force. In addition, the combination of PIX with RFP possesses effective antimicrobial activity against multidrug-resistant strains *in vivo* without detected toxicity. Collectively, these results reveal the potential of PIX in combination with RFP as a therapy option for refractory infections caused by Gram-negative pathogens.

**IMPORTANCE** Bacterial resistance has become increasingly serious because of the widespread use and abuse of antibiotics. In particular, the emergence of multidrug-resistant bacteria has posed a serious threat to human public health. Drug repurposing, the process of finding new uses for existing drugs, provide a promising pathway to solve antimicrobial resistance. Compared to the development of novel antibiotics, this strategy leverages well-characterized pharmacology and toxicology of known drugs and is more cost-effective.

## INTRODUCTION

The excessive use of multiple broad-spectrum antibiotics and globalization both in clinical settings and agriculture, have promoted the development of pathogen resistance (1). In particular, the most hazardous pathogens in terms of resistance are listed by the World Health Organization under the acronym “ESKAPE” (Enterococcus faecium, Staphylococcus aureus, Klebsiella pneumoniae, Acinetobacter baumannii, Pseudomonas aeruginosa, and Enterobacter species) (2). These important pathogens are major culprits of nosocomial infection and are able to “escape” the biocidal action of antibiotics, which leads to treatment failure and increased mortality, with an estimated 700,000 deaths globally each year (3). In an alarming contrast, such a crisis is accompanied by a reduction in the development of new FDA-approved antimicrobials over the past 50 years (4) due to the retreat of pharmaceutical enterprises from new antibiotic development. Hence, alternative treatments against drug-resistant pathogens, particularly Gram-negative bacteria, are urgently needed.

Strategies of repurposing approved drugs provide a promising pathway to solve antimicrobial resistance, and the well-characterized pharmacology and toxicology of those drugs can reduce costs of drug development and expedite approval timelines (5). Combination therapies are also a promising strategy to combat drug resistance. For example, antibiotic combinations have been widely used to treat cystic fibrosis-related infections and tuberculosis (6). In addition, some antibiotics in combination with repurposed bioactive compounds exhibited more potent antimicrobial activity. For instance, hydroxyquinoline analog ionophore PBT2, a drug for neurodegenerative disease, resensitized drug-resistant Gram-negative pathogens to polymyxin class antibiotics (7). Another example, the antidiabetic drug metformin, potentiates the antibacterial effect of tetracycline, especially doxycycline and minocycline, against multidrug-resistant S. aureus, E. faecalis, E. coli, and Salmonella enteritidis (8). These results reveal that combinations of nonantimicrobial drugs with conventional antibiotics are a larger space waiting to be explored than monotherapy. Combination therapy can target multiple pathways to facilitate synergy and enable the reduction of a single drug dosage to reach an effective concentration and could also decrease the risk of drug toxicity and increase patient tolerability (9). In addition, combination therapies can also suppress or delay the occurrence of resistant mutations (10).

Pixantrone (PIX), a novel antitumor drug, is a derivative of aza-anthracenedione anthracycline, which lacks the quinone-hydroquinone site responsible for iron binding, thereby reducing the toxicity associated with both anthracyclines and anthracenediones (11). In addition, insertion of a nitrogen heteroatom in the anthracenedione chromophore of PIX leads to extra hydrogen bonding and basic sites, thus augmenting the affinity for DNA (12). Prior studies demonstrated the mode of action of PIX in tumor cell lines, including the formation of DNA adducts, stabilization of a covalent topoisomerase II-DNA complex and stimulation of Topo II-mediated DNA cleavage (13–15). PIX has already passed clinical studies and received approval by the European Medicines Agency as monotherapy for aggressive B-cell non-Hodgkin’s lymphoma (16). Although the antitumor effect of PIX has been well studied, its potential applications in treating bacterial infections have not yet been explored.

Gram-negative pathogens show intrinsic resistance to many antibiotics, due to the barrier effect of their envelope and the broad-spectrum efflux pumps on membrane (17). The selective effect of outer membrane protects bacterial cells from lipophilic and large size antibiotics, including rifampicin, macrolides, and glycopeptides (18). The aim of this study was to find a novel antibiotic adjuvant, which was capable to restore the activity of disused antibiotics against problematic Gram-negative pathogens. To identify potential potentiators, a library containing 2049 FDA-approved compounds was tested in combination with rifampin (RFP), a well-tolerated antibiotic with a definite mechanism, against type strains of A. baumannii, K. pneumoniae and P. aeruginosa. Among the compounds screened, we selected PIX for more in-depth study. We performed a variety of experiments to explore the potency of PIX and to elucidate the underlying mechanism.

## RESULTS

### PIX enhanced the antimicrobial activity of RFP against Gram-negative pathogens.

**FIG 1:**
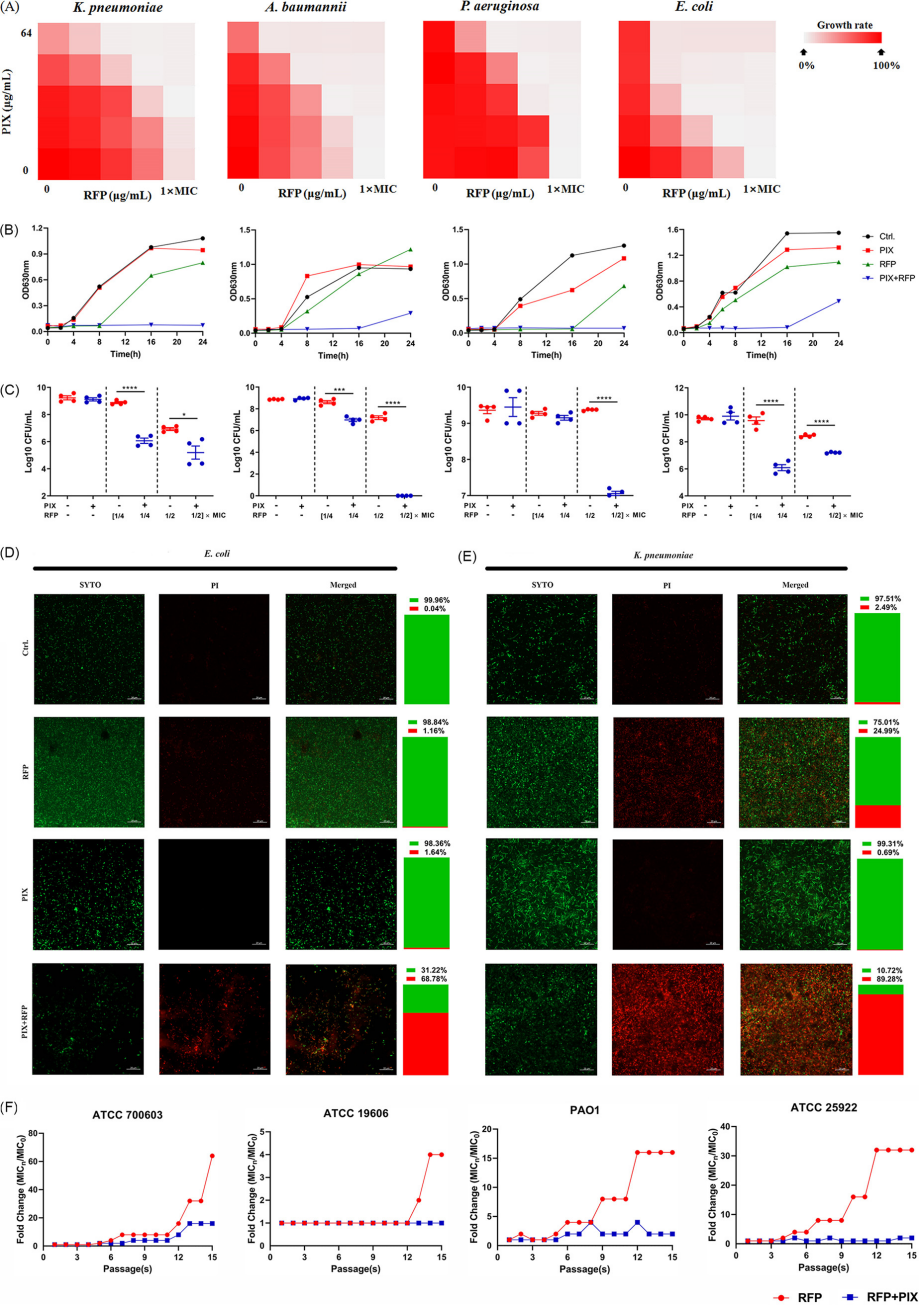
Effects of PIX on the antimicrobial activity of RFP against Gram-negative bacteria. (A) Combinational antimicrobial effects between PIX and RFP against K. pneumoniae ATCC 700603, A. baumannii ATCC 19606, P. aeruginosa PAO1 and E. coli ATCC 25922 by checkerboard assay. The MICs of RFP for four type strains are 64, 4, 64 and 32 μg/mL, respectively (B) The time-growth curve of four type strains treated with PIX and RFP alone or in combination (16 μg/mL + 1/2 ×MIC for ATCC 700603; 32 μg/mL + 1/8 ×MIC for ATCC 19606; 16 μg/mL + 1/2 ×MIC for PAO1; 16 μg/mL + 1/4 ×MIC for ATCC 25922). (C) Viable cell counting of bacteria after exposure to PIX (32 μg/mL) and RFP (1/4 × MIC or 1/2 × MIC) alone or in combination for 16 h. Data are presented as mean ± SD and the significances were determined by one-way ANOVA (*, *P < *0.05; **, *P < *0.01; ***, *P < *0.001; ****, *P < *0.0001). Representative CLSM images of (D) E. coli ATCC 25922 and (E) K. pneumoniae ATCC 700603 treated with PIX (32 μg/mL) and RFP (1/4 × MIC) alone or in combination and stained with SYTO9/PI to detect cell viability. Scale bar: 20 μm. (F) Resistance development during serial passaging of bacterial strains in the presence of RFP alone or RFP coupled with PIX (32 μg/mL).

By using the type strains A. baumannii ATCC 19606, K. pneumoniae ATCC 700603 and P. aeruginosa PAO1, we screened the synergistic antimicrobial activity between RFP, an antibiotic with a definite mechanism, and an FDA-approved library with 2046 compounds by a simple bacterial growth inhibition assay (Fig. S1). The primary screening identified 116 hits that had synergistic activity with RFP. After excluding well-studied compounds, 26 hits (Fig. S2) were further confirmed by a checkboard assay. Among these hits, PIX was found to exhibit the most potent synergistic effect against these pathogens, although PIX displayed no direct antibacterial activity by itself with an MIC > 64 μg/mL (Fig. 1A). Thus, we focused on the potential of PIX as an RFP adjuvant in subsequent studies. Furthermore, the synergistic potential antimicrobial activity between PIX and RFP was verified by a time-growth assay. During a 24-h exposure period, the combination of sub-MIC PIX with sub-MIC RFP displayed obvious synergistic growth inhibition against the tested Gram-negative type strains (Fig. 1B). Consistently, 32 μg/mL PIX notably enhanced the antimicrobial activity of RFP and reduced the number of viable cells against these strains after 16 h of treatment (Fig. 1C). By Live/Dead fluorescent staining, the number of viable bacteria in the combined treatment group was obviously lower than that in the control group or the single-use group for both E. coli (Fig. 1D) and K. pneumoniae (Fig. 1E). To explore the inhibitory role of PIX on the resistance development induced by RFP, a sequential passaging resistance assay was conducted with sub-MICs of RFP in the presence or absence of PIX for 15 days. As shown in Fig. 1F, PIX largely reduced the resistance occurrence induced by RFP compared to RFP alone.

Next, the combination therapy was also verified in drug-resistant clinical isolates. Consistent with the above observation, PIX effectively enhanced RFP activity against hard-to-treat superbugs (XDR or PDR strains) with a 2- to 16-fold reduction in MICs (Table 1). In addition, synergy was also observed between RFP and polymyxin B. However, PIX exhibited no synergistic activity with other antibiotics (Fig. S3). Furthermore, the combination of PIX with RFP did not have a synergistic effect against Gram-positive bacteria (Fig. S4).

**TABLE 1:** PIX enhance the antimicrobial activity of RFP against multidrug-resistant strains

Strains	Resistant pattern	MIC (μg/mL)			Fold change^*a*^	MBC (μg/mL)			Fold change^*b*^
		PIX	RFP	RFP+ PIX (32 μg/mL)		PIX	RFP	RFP+ PIX (32 μg/mL)	
K. pneumoniae
KPWANG	XDR^*c*^	>128	16	4	4	>128	32	16	2
KPLUO	XDR^*c*^	>128	16	2	8	>128	32	8	4
LH2020	PDR^*d*^	>128	32	8	4	>128	64	16	4
A. baumannii
AB1069	XDR^*c*^	>128	8	2	4	>128	16	4	4
AB1208	XDR^*c*^	>128	8	2	4	>128	16	8	2
AB2730	MDR^*e*^	>128	4	<0.25	>16	>128	64	16	4
P. aeruginosa
PA1	XDR^*c*^	>128	16	8	2	>128	64	16	4
PA2	XDR^*c*^	>128	64	16	4	>128	128	16	8
E. coli
Y0064	XDR^*c*^	>128	16	8	2	>128	64	8	8
Y9395	XDR^*c*^	>128	16	4	4	>128	64	4	16
Y9592	XDR^*c*^	>128	16	4	4	>128	64	8	8
Y9633	XDR^*c*^	>128	16	4	4	>128	32	4	8

a Fold change of MIC for RFP = MIC_(RFP)_/MIC_(RFP+PIX)_.

b Fold change of MBC for RFP = MBC_(RFP)_/MBC_(RFP+PIX)_.

c XDR, Extensively drug resistant strain.

d PDR, Pandrug-resistant strain.

e MDR, Multidrug-resistant strain.

### PIX interacted with the bacterial cell outer membrane.

**FIG 2:**
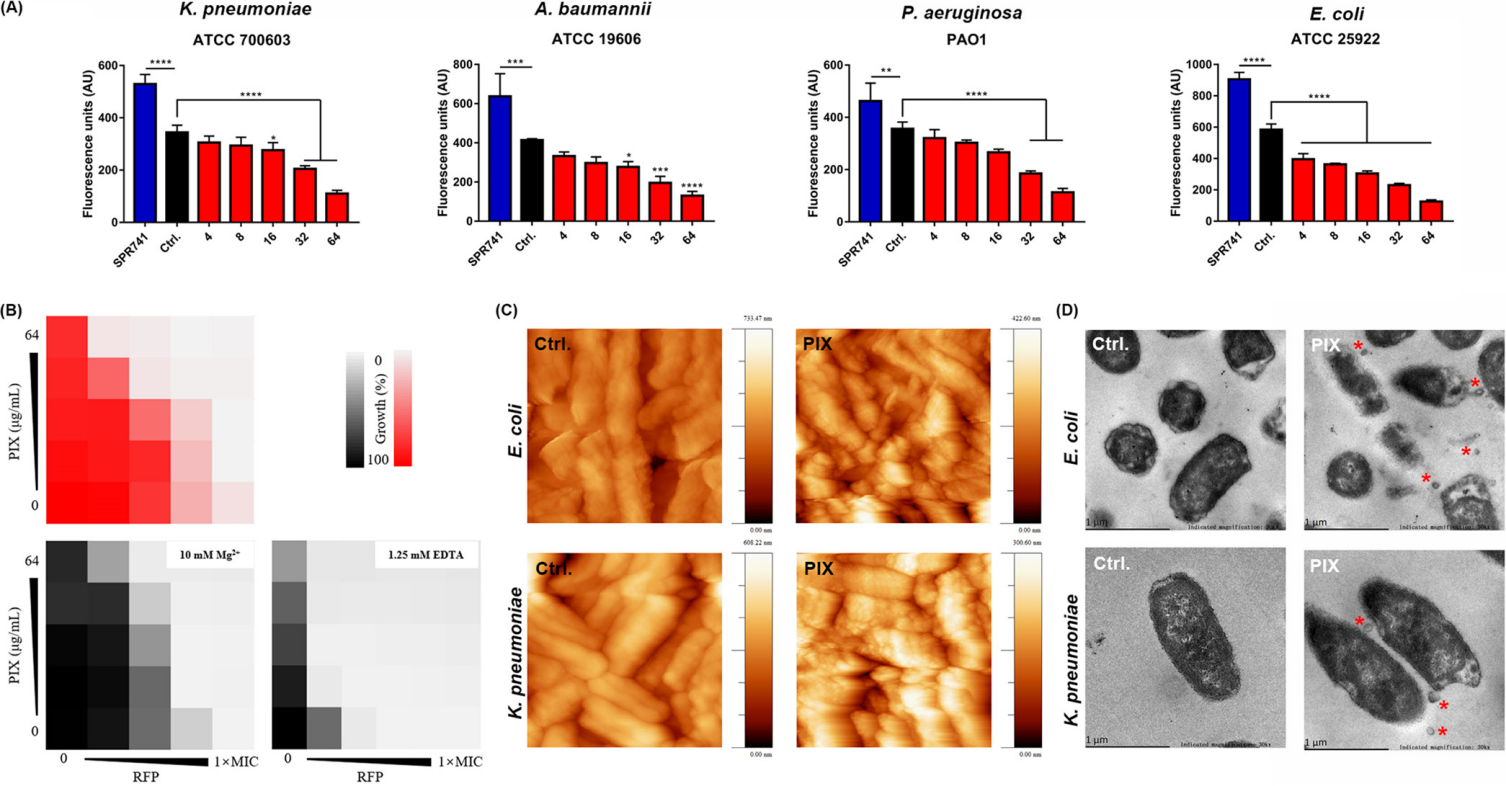
The interaction of PIX on bacterial cell outer membranes. (A) Outer membrane permeability was evaluated by NPN after treatment with either increasing concentrations of PIX or SPR741 (positive control, 16 μg/mL). (B) Effects of exogenous Mg^2+^ (10 mM) and EDTA (1.25 mM) on the synergy between PIX and RFP against E. coli ATCC 25922. The top panel shared the same image as the rightmost panel of Fig. 1A due to the same treatment. (C) AFM and (D) TEM images of E. coli ATCC 25922 and K. pneumoniae ATCC 700603 incubated with PIX (32 μg/mL) or 0.1% DMSO (control). Red triangles indicate bacteria forming extracellular vesicles.

Having shown that PIX potentiates the bactericidal activity of RFP against Gram-negative pathogens, we next sought to elucidate its potential mechanisms. First, no cytoplasmic membrane-disrupting activity was observed in the presence of various concentrations of PIX by staining with the fluorescence probes SYTOX Green, PI, and DiSC3(5) (Fig. S5). Subsequently, we measured the disrupting effect of PIX on the permeability of the outer membrane by the hydrophobic fluorescent probe NPN. Interestingly, after treatment with varying concentrations of PIX, these tested strains showed a dose-dependent decrease in fluorescence intensity (Fig. 2A), which may be related to morphological changes in the bacterial membrane. However, the specific mechanism is unknown. Although supplementation with sub-MICs of Mg^2+^ (an outer membrane strengthener) (Fig. S6) only slightly reduced the synergistic potentiation of PIX to RFP, sub-MICs of EDTA (an outer membrane abolishing reagent) (Fig. S6) obviously potentiated their synergistic effect (Fig. 2B). To better understand the activity of PIX on bacterial surface morphology, atomic force microscopy (AFM) was performed. Interestingly, the surface topography of PIX-treated cells was characterized by strong undulations, whereas untreated bacteria remained largely uniform (Fig. 2C and Fig. S7). Additionally, transmission electron microscopy (TEM) showed that PIX-treated cells presented ultrastructural damage in forming extracellular vesicles, whereas untreated cells exhibited normal and clear structures (Fig. 2D). These results indicate that PIX could interact with bacterial cell outer membranes.

### PIX induced irreversible reactive oxygen species and reactive nitrogen species damage.

**FIG 3:**
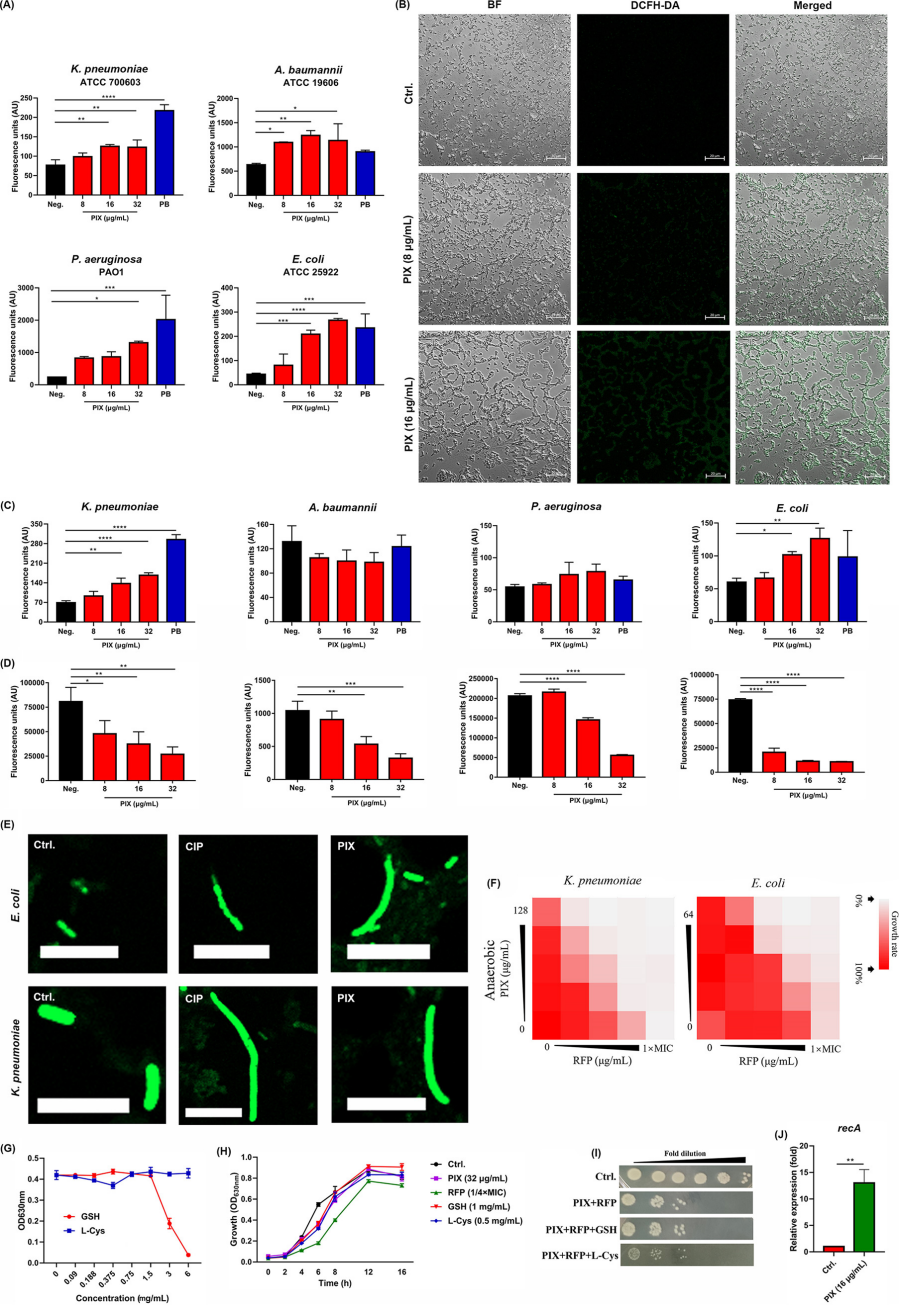
Effect of PIX on bacterial redox homeostasis. (A) K. pneumoniae ATCC 700603, A. baumannii ATCC 19606, P. aeruginosa PAO1 and E. coli ATCC 25922 were treated with varying concentrations of PIX or positive control (PB, polymyxins B, 16 μg/mL), and the ROS level was measured by DCFH-DA. (B) DCFH-DA staining of E. coli ATCC 25922 visualized by CLSM. (C) RNS levels of bacteria treated with PIX or PB (16 μg/mL) were quantified by DAF-FM DA. (D) Intracellular ATP level detection in four type strains treated with varying concentrations of PIX (8-32 μg/mL). (E) Representative CLSM images of E. coli ATCC 25922 and K. pneumoniae ATCC 700603 following treatment with PIX (32 μg/mL) or CIP (10 × MIC). Scale bar: 20 μm. (F) Checkerboard assays of PIX and RFP against E. coli ATCC 25922 and K. pneumoniae ATCC 700603 under anaerobic conditions. (G) The antimicrobial susceptibility of GSH and L-Cys against E. coli ATCC 25922. (H) The growth of E. coli ATCC 25922 supplemented with either PIX (32 μg/mL), RFP (1/4 × MIC), GSH (1 mg/mL) or L-Cys (0.5 mg/mL). (I) E. coli ATCC 25922 was treated with a combination of PIX and RFP supplemented with or without antioxidants (GSH, 1 mg/mL or L-Cys, 0.5 mg/mL), and the cultures were plated after incubation for 6 h. (J) The SOS-associated gene *recA* expression of E. coli ATCC 25922 in the presence of PIX (16 μg/mL) was determined by qRT-PCR.

Reactive oxygen species (ROS) are responsible for maintaining redox homeostasis in bacteria, and destruction of the cellular oxidative equilibrium may induce cell death (19). Hence, we detected the total ROS level by using the DCFH-DA probe and found that PIX triggered the accumulation of ROS in a dose-dependent manner in the type strains and XDR strains (Fig. 3A and Fig. S8). Consistently, as visualized by laser confocal microscope (CLSM) observation, an increase in fluorescence intensity in PIX-treated bacteria was found (Fig. 3B). It was previously reported that oxidative stress can cause DNA damage by inducing hydroxyl radicals (20), which is consistent with our observation that the SOS response-related gene *recA* was also activated by PIX (Fig. 3J). Reactive nitrogen species (RNS) are toxic molecules produced when nitric oxide (NO) encounters ROS (21). Subsequently, the effect of PIX on total RNS was measured by the DAF-FM DA probe. As we expected, the fluorescence intensity was largely increased in the presence of PIX in a dose-dependent manner in K. pneumoniae ATCC 700603 and E. coli ATCC 25922 (Fig. 3C). As we expected, we failed to obtain such a result in A. baumannii ATCC 19606 and PAO1, since they are obligate aerobes that only utilize oxygen, not NO. As aerobic energy production is severely impeded during nitrosative stress (22), the intracellular levels of ATP also significantly decreased in a dose-dependent manner in the tested strains treated with PIX (Fig. 3D). Previous studies have reported that fluoroquinolones such as ciprofloxacin (CIP) elevate the levels of intracellular ROS and result in filamentous bacteria (23, 24). Similarly, elongated bacterial cells in E. coli ATCC 25922 and K. pneumoniae ATCC 700603 (Fig. 3E) after incubation with PIX were observed. In addition, we found that PIX still exhibited synergy with RFP even under anaerobic conditions (Fig. 3F). Even with the addition of sub-MICs of antioxidants glutathione (GSH) or l-cysteine (L-Cys, Fig. 3G and H), no detriment on the synergistic effect was found (Fig. 3I), suggesting that the ROS/RNS inducing ability by PIX was irreversible.

### Proton motive force disruption by PIX.

**FIG 4:**
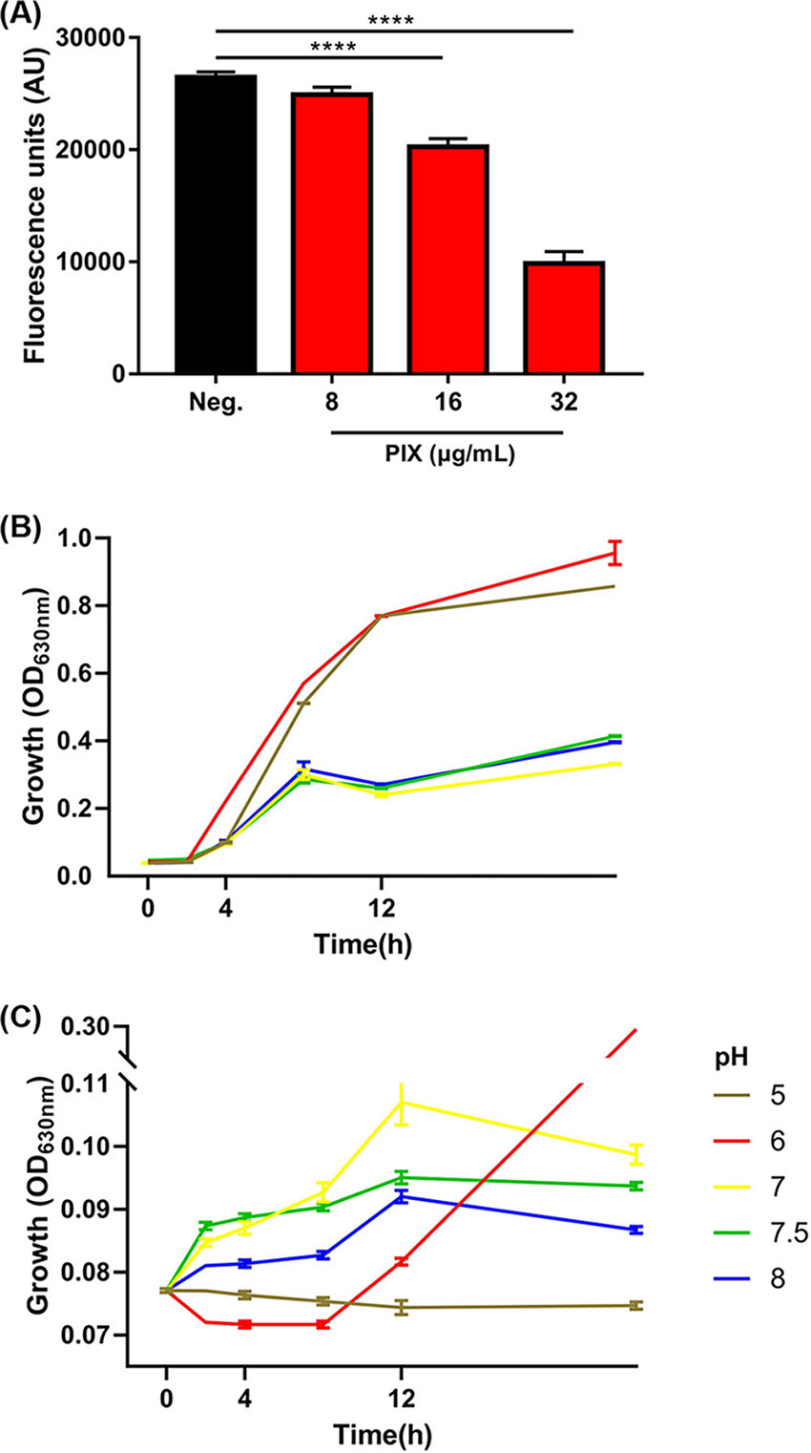
The effect of PIX on bacterial PMF. (A) Detection of the intracellular pH of E. coli ATCC 25922 by monitoring the fluorescence intensity of BCECF-AM. (B) The growth of E. coli in pH-adjusted broth. (C) Decreased antibacterial activity of the combination of RFP and PIX in H^+^-reduced medium.

Given that proton motive force (PMF), an electrochemical gradient of protons, is essential for maintaining bacterial processes, we next sought to elucidate whether PIX causes disruption in PMF. Hence, we used BCECF-AM to measure ΔpH, a key component of PMF (25), and PIX led to decreased fluorescence in a dose-dependent manner in E. coli ATCC 25922 (Fig. 4A) and other Gram-negative pathogens including XDR isolates (Fig. S9), indicating a remarkable reduction in the internal pH value. Furthermore, we determined the synergistic activity between PIX and RFP against E. coli in the presence of pH-adjusted MH broth ranging from 5~8, since increasing extracellular pH leads to a decrease in ΔpH. PIX showed better activity under acidic conditions (Fig. 4B and C), where the PMF is mainly maintained by ΔpH (26). These results suggest that PIX disrupted PMF by affecting the bacterial intracellular pH. Previous studies demonstrated that dissipating the PMF can enhance the antibacterial effect of doxycycline (27) and eliminate the sterilization effect of aminoglycoside antibiotics (28). Consistent with our results, a synergistic effect between PIX and doxycycline was found (Fig. S10A), whereas PIX and kanamycin were antagonistic (Fig. S10B). Considering that PMF is critical for the functions of flagellar (29), we detected the effects of PIX on bacterial swimming motility. When treated with PIX, the swimming diameter was significantly inhibited in E. coli and P. aeruginosa (Fig. S11), indicating that motility was suppressed due to PMF collapse.

### Other underlying mechanisms of PIX.

**FIG 5:**
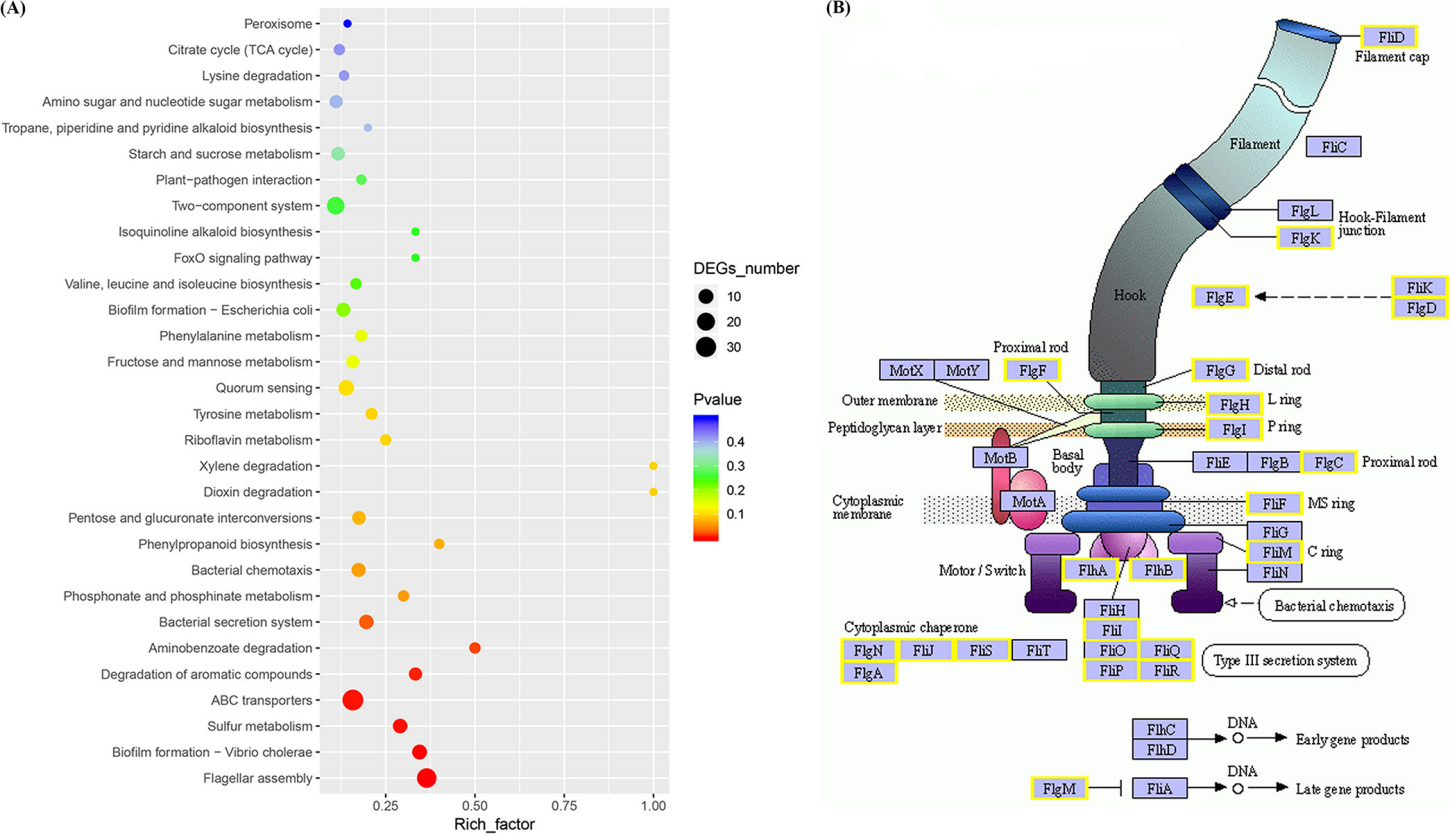
Transcriptome alterations in E. coli ATCC 25922 treated with PIX. (A) KEGG enrichment analysis of the DEGs in E. coli after exposure to PIX (16 μg/mL) for 1 h. (B) Scheme of genes involved in flagellum assembly, quoted from the KEGG database. Yellow represents genes affected by PIX.

To further validate the molecular mechanism of PIX, we conducted transcription analysis of E. coli ATCC 25922 treated with PIX for 1 h. A total of 488 differentially expressed genes (DEGs) were identified between the PIX group and the control group. KEGG enrichment analysis revealed that these DEGs were involved in flagellar assembly, biofilm formation, ABC transporters, the bacterial secretion system and metabolism-related pathways (Fig. 5A). It is plausible that PIX disrupted cellular redox balance by downregulating oxidoreductase (including *soxS*, *nifJ*, *fpr*, etc.) and TCA cycle-associated genes (*PorA*, *Mqo*, etc.), and resulted in the accumulation of ROS and RNS (Table S3). Notably, flagellar assembly and ABC transporter-related genes drastically decreased (Fig. 5B and Table S3), indicating weakened functions of flagellar motility and efflux pumps, which was consistent with PMF collapse by PIX (Fig. 4).

### *In vivo* synergistic antimicrobial activity between PIX and RFP.

A critical concern for combination therapy is whether there is superimposed toxicity. Thus, hemolysis and cytotoxicity of PIX to mammalian cells were detected. Even when used at high concentrations up to 128 μg/mL, PIX only showed negligible hemolysis to human red blood cells (RBCs) (Fig. S12A). Similarly, PIX was also extremely slightly toxic to BEL-7404, RAW264.7, HMC3, HK-2 and U251 cell lines, even at a concentration of 32 μg/mL (Fig. S12B to F), although obvious toxicity to renal carcinoma 786-O cells was found (Fig. S12G). Collectively, although PIX exhibited certain dose-dependent toxicity to tumor cells, its toxicity to normal cells was negligible. It was previously reported that the blocking effect of drugs on the I_Kr_ current is usually attributed to cardiac arrhythmia (30). Thus, manual patch-clamp methods were performed on BEL-7404 cells to replace I_Kr_ in cardiomyocytes. PIX did not influence the action potential duration with an IC50 > 60 μM (Fig. S12I), while cisapride blocked (positive control) the hERG channel with an IC50 of 14.3 nM (Fig. S12H).

Although moderate cytotoxicity was found at the concentration of 128 μg/mL, PIX only showed no or slight apoptosis-inducing effects against RAW264.7 cells at the antimicrobial concentrations by CLSM observation (Fig. S13). Flow cytometry analysis showed that PIX showed no apoptosis-inducing effects against LO2 and HepG2 cells even at a concentration of 32 μg/mL (Fig. S14A to D). Notably, no promotional effect on ROS generation in mammalian cells, including HepG2 and LO2 cell lines (Fig. S14E to H and Fig. S15), is similar to the previous notion that PIX lacks redox activity in the human myocardium (31). Subsequently, we detected the housekeeping gene-expressed proteins actin and tubulin in LO2 cells. The expression of actin and tubulin both remained invariant in the cells treated with PIX, as expected (Fig. S14I to J).

For *in vivo* toxicity, the tolerance to a high dose of PIX was measured by administering a single dose of PIX (0, 60, 100, 200 or 400 mg/kg). As shown in Fig. S16A, all the mice were well tolerant to administration of 100 mg/kg PIX. Consistently, we did not observe enhanced toxicity in the combinational treatment by PIX and RFP for any of the hematological parameters (Fig. S16B) or cardiac/renal/hepatic functional biomarkers (Fig. S16C to E). Similarly, no significant pathological changes in the main organ tissues (heart, liver, spleen, lung, and kidney) were observed by histological analysis (Fig. S16F). Taken together, these data indicate that the joint use of PIX and RFP is safe and well tolerated *in vivo*.

**FIG 6:**
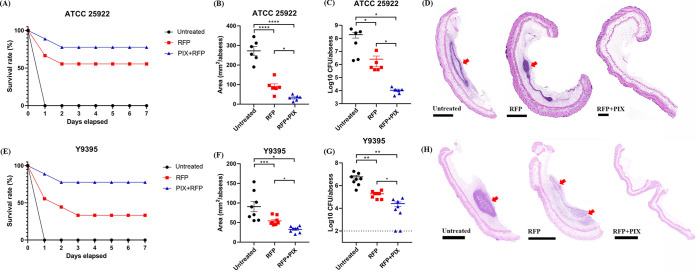
*In vivo* antimicrobial efficacy of combination therapy. The combination of PIX and RFP increased the survival rate of mice infected with (A) E. coli ATCC 25922 or (E) XDR E. coli Y9395 during 7 days postinfection in a peritonitis-sepsis model. The related abscess area (B and F), bacterial load quantification (C and G) and H&E staining of the abscess (D and H) are shown. Scales, 200 μm. Untreated (saline); RFP (20 mg/kg); PIX + RFP (30 + 20 mg/kg).

Next, the *in vivo* antimicrobial efficacy was evaluated in both systemic infection models and abscess models. In the mouse peritonitis-sepsis model, mice infected with E. coli ATCC 25922 or XDR E. coli Y9395 all died within 24 h. However, the combination therapy resulted in 80% survival, which was significantly higher than that obtained by RFP monotherapy (Fig. 6A and E). This survival advantage was then validated in a murine subcutaneous abscess model by ATCC 25922. The combination of PIX and RFP significantly reduced the abscess area and viable bacterial loads in the abscess compared with the single treatment group (Fig. 6B and C). Consistently, H&E analysis showed that the combination therapy group had significantly reduced inflammatory infiltration in the abscess (Fig. 6D). For infection with XDR Y9395, similar synergistic antimicrobial efficacy was found by both abscess area and CFU counting (Fig. 6F and G). The combination therapy between PIX and RFP only exhibited minimal inflammatory cell infiltration in the dermis and subcutaneous tissue (Fig. 6H). These results demonstrated the *in vivo* adjuvant potential of PIX with RFP to combat resistant bacterial infections.

## DISCUSSION

**FIG 7:**
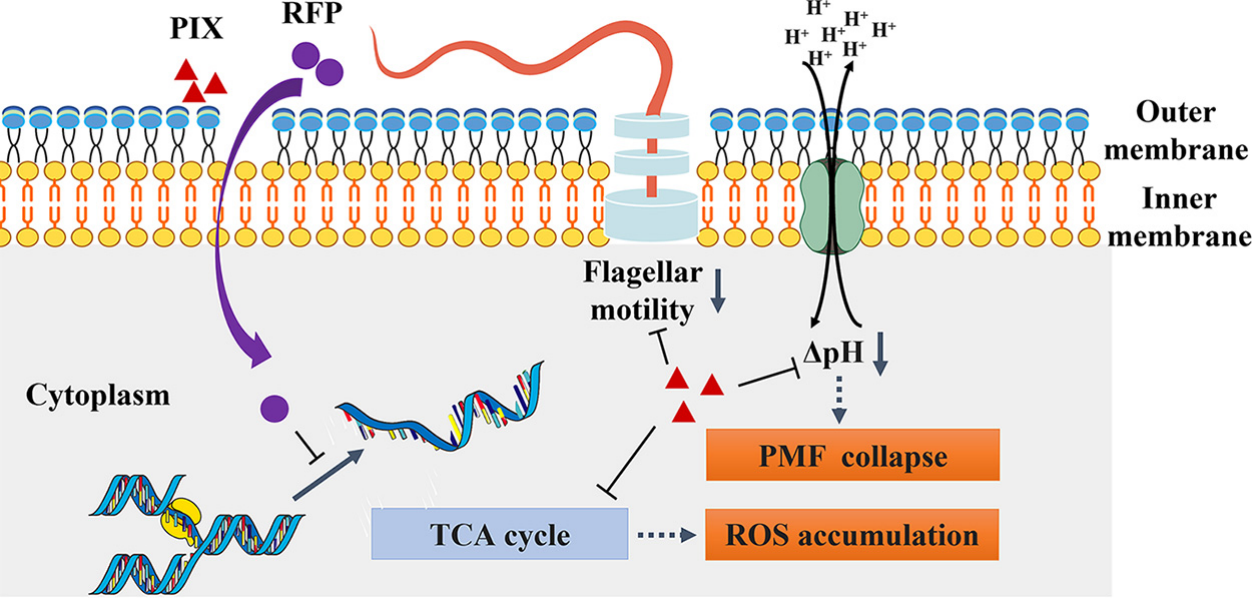
Schematic diagram of the possible mechanism employed by PIX to enhance the antimicrobial effect of RFP. PIX interact with bacterial outer membrane facilitating entrance of RFP, influence the assembly of flagella, induce ROS accumulation and disrupt proton motive force.

In this study, PIX was found to exhibit apparent synergistic antimicrobial potentiation with RFP against Gram-negative pathogens. The synergy is independent of bacterial species and resistance patterns. In a mechanistic study, our results revealed that PIX could effectively impact the assembly of flagella, promote the accumulation of intracellular ROS, and disrupt PMF in bacteria (Fig. 7). Consistently, the combination of PIX and RFP also exhibited effective antimicrobial activity with low toxicity *in vivo*. To the best of our knowledge, this is the first study to systematically investigate the potential of PIX as an antimicrobial adjuvant with RFP against bacteria *in vitro* and *in vivo*.

Previous studies have demonstrated that oxidative stress induction is a main mechanism of action of antibiotics and results in bacterial death (32). For example, kanamycin and ampicillin revealed increased lethality to E. coli deficient in alkyl hydroperoxide reductase *ahpC* (33). Since ROS-based antibacterial agents act on multiple targets, such as nucleic acids, lipids, and proteins, these antimicrobials have attracted the attention of researchers. In particular, some novel antimicrobials, which disrupt the redox defenses of bacterial pathogens, are potential to be part of combinatorial therapies for recalcitrant infection (34). Ebselen, an organoselenium-based drug with cryoprotective and anti-inflammatory properties, possesses obvious antibacterial activity against multidrug-resistant S. aureus by inhibiting the enzyme thioredoxin reductase and inducing oxidative stress (35, 36). Allicin, a thiol-reactive compound, decreases the levels of thiol, which defends against ROS and thus promotes the oxidative stress caused by ROS (37). Notably, oxidative stress is directly proportional to the development of nitrosative stress, which can also disrupt essential biological processes of bacteria and amplify the lethality of antibiotics (38). Similarly, in this study, we found that PIX triggered the accumulation of irreversible ROS and RNS in a dose-dependent manner in Gram-negative bacteria (Fig. 3A and C). In addition, bactericidal antibiotics, including quinolones and aminoglycosides, can induce ROS formation in mammalian cells, which may contribute to some of the adverse effects, such as tendinopathy, nephrotoxicity, and ototoxicity (38, 39). However, in this study, we did not observe detectable ROS production in mammalian cells treated with PIX (Fig. S14E to H and Fig. S15).

Considering the evident synergic effects between PIX and RFP even under anaerobic conditions, we speculated that the specific mechanism of PIX may also include PMF disruption, which is essential for maintaining critical bacterial processes such as flagellar motility, ATP synthesis and transport of solutes across the membrane (40). In our study, the ATP synthesis disorder and motility inhibition induced by PIX also provide evidence to verify our speculation. In addition, PMF is involved in many resistance mechanisms. For example, it is now widely accepted that efflux systems contribute to antibiotic resistance in Gram-negative bacteria and that PMF is critical for the functions of efflux pumps (41, 42). Active maintenance of PMF was previously reported to correlate with the expression of phenotypic antibiotic tolerance (commonly known as persisters) in various bacterial species (43). Similarly, numerous compounds dissipating PMF were reported to exhibit potent bactericidal ability against antibiotic-tolerant cells (44–46). Disrupting the PMF could also prevent bacterial transformation by blocking bacterial competence (47). In another study, it was reported that a nonantibiotic pharmaceutical, melatonin, prevented conjugative transfer between bacterial strains by dissipating PMF (48). Thus, in conclusion, antimicrobial therapy combined with PIX targeting bacterial PMF should be an effective strategy for the treatment of chronic and recurrent resistant bacterial infections.

A previous study reported that no obvious toxicity was found in mice after receiving administration of PIX at 7.5 mg/kg q4d x 3 as an antitumor drug (49), and our data also demonstrated its *in vivo* safety. Moreover, the safety in humans was validated in multicenter, randomized clinical trials (16). For example, in a clinical trial, PIX was well tolerated at a dose of 115 mg/m^2,^ which did not reach the maximum tolerated dose (50). Furthermore, the combined application of PIX and RFP is also an effective strategy to reduce cytotoxicity by decreasing the dosage needed to reach effective antimicrobial activity. In addition, chemotherapy often leads to neutropenia and results in bacterial infection, especially in patients with hematologic malignancies (51). However, as both an antitumor and antimicrobial agent, PIX combined with RFP may be more suitable to treat Gram-negative bacterial infections in patients with lymphoma than monotherapy.

Although, in our study, many experiments have been carried out to demonstrate the potential of PIX to sensitize Gram-negative pathogens to RFP, some knowledge gaps remain unclear. First, our finding of PIX promoting ROS levels and disrupting PMF provides a preliminary mechanism; however, a more in-depth investigation of each synergistic pathway is still required in future work. Second, research to optimize the pharmacologic properties of PIX, such as new chemical analogs, nanoscale carriers and drug delivery systems, is needed, which will also be a focus in our future studies.

## MATERIALS AND METHODS

### Strains and culture conditions.

The strains used in this study are listed in Table S1. Extensively drug-resistant (XDR) A. baumannii isolates of AB1069 and AB1208 and XDR K. pneumoniae strains of KPLUO and KPWANG were kindly provided by Cha Chen (Guangdong Provincial Hospital of Traditional Chinese Medicine, Guangzhou, China). XDR E. coli Y0064, Y9395, Y9592, and Y9633 were kindly provided by Min Li (Shanghai Jiaotong University, Shanghai, China). Other clinical isolates were collected from the Third Xiangya Hospital of Central South University (Changsha, China) and identified using a Vitek 2 Compact (bioMérieux) and matrix-assisted laser desorption/ionization time-of-flight mass spectrometry (Bruker). Gram-positive cocci Staphylococcus and Enterococcus were grown in tryptic soy broth (TSB) (Solarbio). Gram-negative species were grown in Luria-Bertani (LB) broth (Solarbio). All bacteria were propagated at 37°C and 180 rpm. Cation-adjusted Mueller-Hinton II (MH) broth (Solarbio) was used for antimicrobial susceptibility testing.

### High-throughput screening of FDA-approved compounds.

To identify potential antimicrobials, a library containing 2049 FDA-approved compounds was tested against type strains of A. baumannii ATCC 19606, K. pneumoniae ATCC 700603 and P. aeruginosa PAO1 (Fig. S1). The bacteria were suspended in MH broth to ~1 × 10^6^ CFU per milliliter (CFU/mL) with a 1/2-fold MIC of RFP. A total of 99 μL of the suspension was transferred to the wells of a 96-well plate (Corning Costar), and 1 μL of the tested compounds was added to obtain a final concentration of 100 μM. After incubation at 37°C for 16 h, the optical density (OD) at 630 nm was detected using a microplate spectrophotometer (Bio-Rad). Antibiotics and well-studied antimicrobials were excluded, and the first round selected 26 (Fig. S2) hits that were rescreened by checkboard assay. Finally, one active compound (PIX) was chosen for further study (52).

### Antimicrobial susceptibility test.

The MIC of antimicrobials was determined by a standard broth microdilution assay according to the guidelines of the Clinical and Laboratory Standards Institute (53). Briefly, 50 μL of MH medium containing 2-fold diluted compound was mixed with an equal volume of bacterial suspension to approximately 1.0 × 10^6^ CFU/mL into a 96-well plate (Corning Costar). After incubating for 16–18 h at 37°C, the turbidity was measured at OD630, and the lowest drug concentration with no detected bacterial growth was defined as the MIC. To determine the minimum bactericidal concentration (MBC), 10 μL of culture suspension was evenly spread on blood agar plates. After incubating at 37°C for 24 h, the MBC was defined as the minimum drug concentration without bacterial colony growth on the plate.

### Checkerboard assay.

The checkerboard assay was performed to assess the interactions of drug combinations (54). Equal volumes of 2-fold dilutions of PIX and another drug dilution were added vertically and horizontally into 96-well microplates in the presence of ~1 × 10^6^ CFU/mL bacteria. The OD630 was measured after incubation at 37°C for 16–18 h. The fractional inhibitory concentration indices (FICIs) were calculated with the formula FICI = (MIC_A_ in combination/MIC_A_ alone) + (MIC_B_ in combination/MIC_B_ alone). FICI ≤ 0.5 indicates synergy, 0.5 < FICI ≤ 4 indicates no interaction, and FICI > 4 indicates antagonism (55).

### Growth inhibition assay.

The mid log-phase of bacteria was diluted with MH broth in the presence of sub-MICs of PIX and RFP alone or in combination to a final concentration of approximately 10^6^ CFU/mL. The bacterial suspension was incubated at 37°C and 180 rpm. The OD630 of the samples was measured at 0, 2, 4, 8, 16, and 24 h. Viable cells were quantified by CFU counting at 16 h (56).

### Membrane disruption determination by fluorescent probes.

The hydrophobic fluorescent probe 1-*N*-phenylnaphthylamine (NPN) was used to evaluate the outer membrane permeability. Bacteria were grown to an OD630 of 0.5 in LB broth. Cells were harvested by centrifugation, washed, and resuspended in 5 mL of HEPES buffer (pH 7.2). The bacterial suspensions were treated with PIX (4~64 μg/mL) or SPR741 (positive control) in the presence of 10 μM NPN for 15 min (57). Fluorescence intensity was recorded with excitation/emission wavelengths of 350 nm/420 nm by a microplate reader (PerkinElmer EnVision).

Similarly, SYTOX Green (final concentration of 5 μM) (58) and propidium iodide (PI, final concentration of 10 μM) (8) were applied to evaluate the cell membrane integrity with excitation/emission wavelengths of 504 nm/523 nm and 535 nm/615 nm, respectively. In addition, the membrane potential was measured using 3,3-dipropylthiadicarbocyanine iodide (DiSC3[5], final concentration of 2 μM) (59) with an excitation/emission wavelength of 622 nm/670 nm with an interval of 5 min for 30 min.

### Live/dead cell staining.

Bacteria were grown to log-phase and adjusted to ~10^6^ CFU/mL. The bacterial suspension was further incubated with either PIX (32 μg/mL) or RFP (1/4 × MIC) alone or in combination at 37°C and 180 rpm for 8 h. Then, the bacterial suspension was stained with SYTO 9 (10 μM) and PI (10 μM) for 15 min at room temperature in the dark. The morphology was imaged by CLSM (Zeiss LSM 800) with excitation/emission wavelengths of 488 nm/550 nm and 540 nm/620 nm for SYTO 9 and PI, respectively, and analyzed by ImageJ software (60).

### Atomic force microscopy.

Mid-log cultures of E. coli ATCC 25922 and K. pneumoniae ATCC 700603 were treated with PIX (32 μg/mL) or 0.1% DMSO at 37°C and 200 rpm for 1 h, and the bacteria were harvested by centrifugation at 4500 × *g* for 8 min. Then, 20 μL of the bacterial suspension was poured onto a microscope glass slide (Thermo Scientific SuperFrost Microscope slides 76 × 26 mm, with 1 mm thickness) and air-dried under a laminar flow hood at room temperature for 20 min. Glass slides were cleaned with pure ethanol before bacterial deposition. AFM measurements were performed using a Dimension ICON AFM (Bruker) (61).

### Transmission electron microscopy.

For TEM, the samples were processed as described above, and pelleted bacteria were fixed with 1.5 mL of 2.5% glutaraldehyde at 4°C for 24 h. Subsequently, fixed cells were washed with 0.1 M sodium cacodylate buffer and secondarily fixed with 1% osmium tetroxide. After washing with water and maleate buffer, the fixed cells were then substituted in maleate buffer containing 1% uranyl acetate for 1 h. The cells were dehydrated in ethanol by gradient concentrations (30%, 50%, 70%, and 90%) and embedded in Spurr’s low viscosity resin. Ultrathin sections (60 to 80 nm) were cut using a Reichert Ultracut-S microtome, poststained with 2% uranyl acetate followed by lead citrate, and imaged with TEM (HITACHI HT-7700) (62).

### Reactive oxygen species detection.

The levels of ROS in Gram-negative-type strains treated with PIX were detected by a 2′,7′-dichlorofluorescein diacetate (DCFH-DA, Beyotime) probe. Briefly, the strains were grown to an OD630 of 0.5 in LB broth. Cells were pelleted by centrifugation, washed, and resuspended in 5 mL of 1× phosphate-buffered saline (PBS, pH = 7.4). Then, the bacterial suspension was incubated with DCFH-DA (10 μM) for 30 min. After washing twice with PBS to remove excess probes, 90 μL of the probe-labeled cells and 10 μL of PIX (final concentration at 8–32 μg/mL) were mixed in 96-well plates. After incubation for 30 min at 37°C, the fluorescence intensity was measured by a microplate reader at excitation/emission wavelengths of 488 nm/525 nm (63). For CLSM observation, 10 μL of the stained suspension was spread on a sterile slide. After air drying in the dark, images were captured.

### Reactive nitrogen species quantification.

Nitric oxide generation of bacterial strains treated with PIX (8-32 μg/mL) was estimated with the probe 4-amino-5-methylamino-2′,7′-difluorofluorescein diacetate (DAF-FM DA) (64). Briefly, bacteria were grown to an OD630 of 0.2 in LB broth. After washing, bacterial cells were resuspended in HEPES buffer (pH 7.2) and incubated with 10 μM DAF-FM DA for 30 min in the dark. After washing with HEPES buffer to remove excess probes, 90 μL of labeled cells and 10 μL of PIX were mixed in microplates for 30 min. The excitation and emission wavelength settings on the spectrophotometer were 500 and 515 nm, respectively.

### Intracellular ATP detection.

The intracellular ATP levels of bacterial strains were measured by an ATP assay kit (Beyotime). Briefly, the bacteria were grown in LB broth to an OD630 of 0.5. Cells were pelleted, washed, and resuspended in PBS. After treatment with various concentrations of PIX (8 to 32 μg/mL) for 1 h, the bacterial suspension was centrifuged at 4°C to remove the supernatant. Then, the bacteria were lysed with lysozyme, and the supernatant was collected. Detecting reagent and the supernatant were mixed into a microplate, and the luminescence was measured by using a microplate reader (8).

### Quantitative reverse transcription PCR.

Log-phase cultures of E. coli ATCC 25922 were diluted with LB in the presence or absence of 16 μg/mL PIX. After incubation at 37°C and 180 rpm for 8 h, the bacteria were harvested, and the total RNA was extracted using the E.Z.N.A. Bacterial RNA Kit (Omega). Reverse transcription of 1 μg extracted RNA was performed using the TransScript All in-One First-Strand cDNA Synthesis SuperMix (Transgene) following the manufacturer’s protocol. The mRNA levels of *recA* relative to the control genes (16S rRNA) were determined by quantitative reverse transcription PCR (qRT-PCR) with TransStart Tip Green qPCR SuperMix (Transgene) by using the CFX96 Real-Time PCR Detection System (Bio-Rad Laboratories). The primers used are described in Table S2.

### PMF determination by BCECF-AM staining.

The PMF of E. coli ATCC 25922 after treatment with PIX was measured by a pH-sensitive fluorescence probe BCECF-AM. Bacteria were grown to an OD630 of 0.5 in LB broth. Then, the cells were pelleted by centrifugation, washed, and resuspended in 5 mL of HEPES buffer (pH 7.2). BCECF-AM was added to obtain a final concentration of 20 μM. After the fluorescence stabilized, 25 μM glucose (negative control) or varying concentrations of PIX (8-32 μg/mL) were added, and the fluorescence was monitored using a fluorescence spectrometer at an excitation/emission wavelength of 500 nm/522 nm (8).

### PMF detection by pH-adjusted MH broth.

The pH value of the MH broth was adjusted by adding HCl or NaOH to a range from 5.0 to 8.0. Mid log-phase E. coli ATCC 25922 was diluted to 1 × 10^6^ CFU/mL in pH-adjusted broth with or without a series of concentrations of PIX. Then, the bacterial suspension was incubated at 37°C and 180 rpm, and the turbidity was measured by OD630 at 0, 4, 8, 12, and 24 h.

### Transcriptomic analysis.

Mid-log growth phase E. coli ATCC 25922 was diluted in MH broth to ~1 × 10^6^ CFU/mL in the presence of 16 μg/mL PIX or 0.1% DMSO (control). After incubating for 1 h, the cells were harvested by centrifugation, and the total RNA of the samples was extracted using an E.Z.N.A. Bacterial RNA Kit (Omega). rRNA was depleted with a Ribo-Zero Magnetic Kit (Illumina), and the complementary DNA (cDNA) library was prepared using the TruSeqTM Stranded RNA Sample Prep Kit (Illumina). Illumina sequencing was performed using the TruSeqTM SBS Kit (300 cycles) (Illumina) with a read length of 2 × 150 bp. Differential gene expression was analyzed using the fragments per kilobase of transcript per million mapped reads (FPKM) method. Only genes that were significantly (*P* < 0.05) differentially regulated with at least a 2-fold change were considered.

### Swimming motility assay.

The motility plates were freshly prepared with 0.3% (wt/vol) agarose, 0.5% (wt/vol) yeast extract, 0.5% (wt/vol) NaCl, and 1% (wt/vol) tryptone in the presence or absence of PIX (16 μg/mL). The mid log-phase E. coli ATCC 25922 was washed and resuspended in sterilized saline to an OD_600_ of 1.0, followed by inoculating 2 μL of the bacterial suspension onto the center of the plate. After incubating in a humid atmosphere for 16 h at 37°C, the plates were imaged (65). The migration diameters were measured by a caliper.

### Resistance development studies.

To assess the development of bacterial resistance to RFP in the presence or absence of PIX (32 μg/mL), the Gram-negative type strains were sequentially passaged in MH broth with sub-MICs of antimicrobials. Briefly, the MICs for bacteria to RFP with or without PIX were determined by broth microdilution as mentioned above. After overnight incubation, the bacterial suspension grown in the presence of 0.5 × MIC was diluted 1:1000 with MH broth and was further used to determine the MICs by RFP or RFP+PIX. This procedure was repeated consecutively for 15 days in triplicate (66).

### RBC hemolysis and cytotoxicity.

Fresh human RBCs (Hemo Pharmaceutical and Biological Co.) were washed with PBS and diluted to a final concentration of 5% (vol/vol). Then, those cells were treated with PIX at concentrations up to 256 μg/mL at 37°C for 1 h. The supernatant was collected by centrifugation at 1000 × *g* for 5 min, and the absorbance at 570 nm (A570) was measured (67). Triton X-100 (0.1%) and DMSO (1%) were used as positive and negative controls, respectively.

The cytotoxicity of PIX against BEL-7404 (human hepatocarcinoma cell), RAW264.7 (leukemia cells in mouse macrophages), HMC3 (human microglial clone 3 cell), HK-2 (human proximal tubular cell), U251 (human glioma cell), and 786-O (human renal carcinoma cell) cells was determined using the Cell Counting Kit-8 (MedChemEpress) (68). RAW264.7, HMC3, and U251 cells were cultured in Dulbecco’s modified Eagle’s medium (DMEM) supplemented with 10% fetal bovine serum (FBS) and BEL-7404, HK-2 and 786-O cells in RPMI 1640 medium containing 10% FBS. Serially diluted PIX (up to 128 μg/mL) and 1 × 10^4^ cells/well were simultaneously added to 96-well plates. Then, the absorbance was detected at 450 nm (A450) following the manufacturer’s instructions, and the corresponding cytotoxicity was calculated.

### Human ether-a-go-go-related gene (hERG) test.

BEL-7404 cell lines overexpressing human ether-a-go-go-related gene (hERG) were used for the experiment (69). The cultured cells were placed on 35-mm diameter Petri dishes in extracellular solution and incubated at 37°C for 30 min to adhere. A whole-cell patch-clamp method was performed to record hERG currents at room temperature (22°C) using WPC-100 resistive feedback amplifiers (ESF electronic). When filled with internal pipette solution, the resistance of the patch pipettes (GC150F-10; Harvard Apparatus) averaged 2–3 MΩ. Patch pipettes were pulled from borosilicate glass by a P-97 horizontal puller (Sutter Instrument Co.). For drug application, PIX or cisapride (positive control) was applied using a piezoelectric-driven micromanipulator (P-287.70, Physik Instrumente) at a flow rate of 100–200 μL/min, as described previously (70).

### Western blot.

For cytotoxicity assessment, total proteins from LO2 cells treated with PIX (16~32 μg/mL) for 24 h were extracted using a Protein Assay reagent kit (Thermo Fisher Scientific). Then, the samples were separated by 10% SDS-PAGE electrophoresis. Subsequently, the proteins were transferred onto PVDF membranes and blocked with 5% skim milk. The membrane was incubated with the primary antibodies anti-β-actin and anti-β-tubulin, followed by HRP-conjugated secondary antibodies (Abcam). Protein bands were developed and analyzed with Alpha Imager 2200 software (Alpha Innotech Corporation).

### Apoptosis detection by cytometer and CLSM.

Apoptosis of LO2 and HepG2 cells induced by PIX was detected by using Annexin V-FITC detection kits (BD Biosciences). Briefly, cells treated with PIX (16 to 32 μg/mL) for 24 h under standard conditions (DMEM with 10% FBS, 5% CO2, 37°C) were collected by centrifugation and resuspended gently in 100 μL of 1× Annexin V binding buffer at a concentration of 1 × 10^6^ cells/mL. Annexin V-FITC (5 μL) and PI (5 μL) were added to the suspension. After incubating at room temperature for 15 min in the dark, the stained cells were analyzed by flow cytometry (71).

For CLSM observation, RAW264.7 cells were cultured in standard conditions (DMEM supplemented with 10% FBS, 5% CO2, 37°C) supplemented with PIX (32 to 128 μg/mL) in humidified incubators. After incubating for 24 h, the cells were collected and stained with Annexin V-FITC detection kits. Then, 20 μL of the suspension was moved onto a glass slide, allowing it to be air dried and imaged.

### Animal preparation.

6- to 8-week-old female ICR mice were purchased from Hunan SJA Laboratory Animal Co. (Changsha, China). Mice were housed in a pathogen-free mouse colony under standardized environmental conditions (temperature = 23 ± 2°C; relative humidity = 55% ± 10%; lighting cycle: 12 h/day) with free access to food and drinking water. Mice were allowed to adapt for 1 week prior to the experiment. All procedures were conducted in accordance with the Guide for the Care and Use of Laboratory Animals and approved by the Ethics Committee of the Third Xiangya Hospital of Central South University (ID: 2021sydw0245). Unless otherwise stated, we used at least 6 (*n* = 6) mice per treatment condition.

### Time-survival curve determination.

The mouse peritonitis infection model was used (8). Mice were relocated randomly to the treatment or control group (*n* = 9 per group). The bacterial inoculum was prepared from an overnight culture of E. coli ATCC 25922 or XDR E. coli clinical isolate (Y9395) and reconstituted in saline. The mice were injected via intraperitoneal (i.p.) injection with a lethal dose of bacterial suspension with 5% mucin (containing ~5 × 10^7^ CFU per mouse). At 2 h postinfection, mice were treated with a single dose of DMSO or RFP (20 mg/kg) or a combination of RFP with PIX (20 + 30 mg/kg) via i.p injection. The survival rates of the treated mice were recorded every 12 h for 7 days.

### *In vivo* abscess model.

The skin and soft tissue infection model was established as described previously (72) with minor modifications. The bacterial inoculum was prepared from an overnight culture of E. coli ATCC 25922 or Y9395 and resuspended in saline. The hair on the back of ICR mice was shaved by an electric razor, and a bacterial suspension (50 μL) containing 1 × 10^7^ CFU was injected into the dorsum subcutaneously (s.c.). Two hours postinoculation, the infected mice were randomly divided into 3 groups and received a single dose of treatment by s.c. (Group 1: saline. Group 2: 20 mg/kg RFP. Group 3: 20 mg/kg RFP with 30 mg/kg PIX). At 24 h postinfection, the areas of the abscesses were measured, and the infected tissues were fixed in 4% paraformaldehyde for hematoxylin-eosin (H&E) staining. Meanwhile, to quantify bacterial loads in the abscesses, the infected skins were excised aseptically and homogenized in saline. The homogenate was serially diluted in sterile saline and spread onto blood agar plates. After incubating at 37°C for 24 h, the number of CFU per abscess was calculated.

### *In vivo* toxicity.

To assess the ultimate limit tolerance of the mice against a high dose of PIX, ICR mice were randomly divided into 5 groups (*n* = 5), and each group was administered a single dose of PIX at different concentrations (0, 60, 100, 200, or 400 mg/kg) via i.p. injections, and survival was monitored every 12 h after administration for a total of 7 days.

The mice were randomly divided into 3 groups (*n* = 5) and i.p. injected with DMSO, PIX (30 mg/kg), or a combination of RFP with PIX (20 + 30 mg/kg). At 24 h postadministration, blood samples of treated mice were collected to detect hematological parameters and cardiac/renal/hepatic functional biomarkers. Meanwhile, the organs of the heart, liver, spleen, lung, and kidney of those mice were excised aseptically for H&E analysis.

### Statistical analysis.

Experiments were independently performed in triplicates. All data were analyzed by GraphPad Prism 8.0 software and are presented as the mean ± standard deviation (SD). To examine the significant differences between groups, the data were compared using Student's *t* test, whereas data comparisons across more than 2 groups were performed using one-way ANOVA followed by Dunn’s multiple comparison test. *P < *0.05 was considered statistically significant. *, *P < *0.05; **, *P < *0.01; ***, *P < *0.001; ****, *P < *0.0001.

### Data availability.

The data that supports the findings of this study are available in the article/supplementary material. Transcriptomic analysis data have been deposited in the National Center for Biotechnology Information’s Sequence Read Archive with accession number PRJNA887829.
