# Supplementary material for: Pixantrone Sensitizes Gram-Negative Pathogens to Rifampin
Source: Microbiol Spectr. 2022 Nov 1;10(6):e02114-22. doi: 10.1128/spectrum.02114-22 (PMC9769682; doi:10.1128/spectrum.02114-22)

Table S1. Bacterial strains used

Strains	Resistant pattern	Source
**Gram-negative**		
E. coli		
ATCC 25922	Type strain, Non-MDR	ATCC
Y0064	XDR	In this study
Y9395	XDR	In this study
Y9592	XDR	In this study
Y9633	XDR	In this study
K. pneumoniae		
ATCC 700603	Type strain, Non-MDR	ATCC
KPWANG	XDR	She et al.
KPLUO	XDR	She et al.
LH2020	PDR	She et al.
A. baumannii		
ATCC 19606	Type strain, Non-MDR	ATCC
AB1069	XDR	In this study
AB1208	XDR	In this study
AB2730	MDR	In this study
P. aeruginosa		
PAO1(ATCC 15692)	Type strain, Non-MDR	ATCC
PA1	XDR	In this study
PA2	XDR	In this study
**Gram-positive**		
S. aureus		
USA300	Type strain, MRSA	Li et al.
E. faecalis		
ATCC 29212	Type strain, Non-MDR	ATCC

ATCC, American Type Culture Collection

References

[1] She P, Liu Y, Xu L, et al. SPR741, Double- or Triple-Combined With Erythromycin and Clarithromycin, Combats Drug-Resistant *Klebsiella pneumoniae*, Its Biofilms, and Persister Cells. *Front Cell Infect Microbiol*. 2022; 12: 858606. doi:10.3389/fcimb.2022.858606

[2] Li Y, She P, Xu L, et al. Anti-hepatitis C virus drug simeprevir: a promising antimicrobial agent against MRSA. *Appl Microbiol Biotechnol*. 2022; 106(7): 2689-2702. doi:10.1007/s00253-022-11878-2

Table S2. Primers used in this study.

Gene	Towards	Sequence (5'→3')	Product length
16s rRNA	Forward	CACACTGGAACTGAGACAC	189 bp
	Reverse	CTTCTTCTGCGGGTAACG	
recA	Forward	CGACTCTCACATGGGCCTT	173 bp
	Reverse	AGAATTTCAGCGCGTTACCAC	

Table S3. Significant expressed DEGs

GeneID	Ctrl-rep1	Ctrl-rep2	PIX-rep1	PIX-rep2	LogFC	Type	p-value	Function
KPHS_01190	0.27	0.36	1.91	0.71	-3.74	up	6.80E-04	transcriptional activator protein
KPHS_01300	17.21	12.22	31.60	28.07	-2.51	up	1.17E-29	transcription termination factor Rho
KPHS_02570	3.04	3.21	0.44	0.65	-2.44	down	1.11E-25	maltose transporter membrane protein
KPHS_02590	6.19	3.98	0.31	0.45	-2.19	down	2.47E-40	maltose/maltodextrin transporter ATP-binding protein
KPHS_02820	1.19	1.91	2.53	4.01	-1.63	up	1.15E-09	excinuclease ABC subunit A
KPHS_03230	2.29	0.77	0.81	0.30	-1.55	down	1.25E-02	formate dehydrogenase-H
KPHS_03730	1.30	0.83	0.39	0.29	-1.46	down	1.92E-05	anaerobic C4-dicarboxylate transporter
KPHS_07520	9.12	9.65	33.47	27.94	-1.35	up	8.04E-71	carbamoyl phosphate synthase small subunit
KPHS_t00220	59.45	43.34	139.22	94.82	-1.26	up	3.24E-10	unknow
KPHS_t00230	49.13	35.41	120.91	81.16	-1.21	up	7.61E-10	unknow
KPHS_09760	0.79	0.70	2.60	2.47	-1.12	up	1.26E-03	xanthine-guanine phosphoribosyltransferase
KPHS_10540	1.36	0.55	0.70	0.13	-1.11	down	2.89E-03	delta-aminolevulinic acid dehydratase
KPHS_10930	21.57	13.03	45.72	29.23	-1.05	up	9.79E-21	putative thiol-alkyl hydroperoxide reductase
KPHS_11440	3.97	2.76	1.25	1.84	-1.02	down	9.56E-03	transcriptional regulator HU subunit beta
KPHS_13690	0.39	0.87	1.65	1.63	1.00	up	1.95E-02	RmbA
KPHS_17650	2.71	2.16	1.21	1.19	1.02	down	1.11E-04	spermidine/putrescine transport system permease component
KPHS_17940	112.76	78.16	274.28	186.23	1.04	up	1.01E-57	translation initiation factor IF-1
KPHS_19260	2.85	0.73	5.76	3.62	1.05	up	5.50E-05	PhoH family protein
KPHS_19620	13.28	10.06	26.91	21.00	1.08	up	2.44E-56	RNase E
KPHS_19670	0.86	1.15	5.47	3.15	1.11	up	1.10E-10	fatty acid/phospholipid synthesis protein
KPHS_20190	2.36	1.17	1.11	0.27	1.14	down	3.93E-03	putative Nudix hydrolase
KPHS_20760	1.49	2.17	0.79	0.98	1.18	down	9.64E-08	protein kinase YeaG
KPHS_29510	2.24	2.36	6.33	3.18	1.19	up	5.48E-06	putative peptide transport protein
KPHS_31180	0.94	0.69	2.29	1.69	1.21	up	1.89E-04	pyruvate kinase
KPHS_32260	1.24	0.47	3.25	1.29	1.22	up	4.44E-03	cation transport regulator
KPHS_32370	71.03	62.46	159.93	142.08	1.26	up	1.28E-148	ribose-phosphate pyrophosphokinase
KPHS_35940	0.31	1.53	0.44	0.33	1.26	down	9.04E-03	peptide ABC transporter ATP-binding protein
KPHS_37080	2.37	1.74	6.33	4.57	1.27	up	1.39E-18	ribonucleotide-diphosphate reductase subunit alpha

KPHS_40570	1.71	0.76	7.24	4.46	1.27	up	2.32E-03	hypothetical protein
KPHS_40580	1.41	0.63	5.47	2.95	1.29	up	5.70E-05	outer membrane protein assembly factor BamE
KPHS_43460	1.66	0.59	4.45	3.29	1.38	up	2.17E-07	MrkB fimbrial protein
KPHS_45810	6.26	5.93	15.61	12.70	1.39	up	5.47E-11	3,4-dihydroxy-2-butanone 4-phosphate synthase
KPHS_46040	1923.41	1695.51	4547.11	3850.98	1.41	up	0.00E+00	30S ribosomal protein S21
KPHS_46250	1.10	0.49	0.31	0.23	1.41	down	1.66E-03	putative glycerol dehydrogenase
KPHS_47950	2.13	1.19	4.51	2.78	1.66	up	1.15E-02	type II 3-dehydroquinate dehydratase
KPHS_48010	7.59	6.41	23.59	20.76	1.67	up	1.37E-38	FMN-linked protein
KPHS_48020	136.68	121.43	458.35	403.49	1.71	up	8.24E-289	DNA-binding protein Fis
KPHS_48780	33.81	32.58	18.66	12.15	1.74	down	1.19E-16	sulfurtransferase complex subunit TusB
KPHS_48830	31.35	21.63	65.27	40.81	1.75	up	4.98E-09	protein SlyX
KPHS_49010	1.62	1.18	8.02	6.36	1.76	up	8.50E-30	MFS transporter TsgA
KPHS_51620	2.12	2.24	5.18	5.25	1.78	up	5.37E-05	guanylate kinase
KPHS_51630	3.06	1.95	6.17	5.93	1.79	up	4.07E-03	DNA-directed RNA polymerase subunit omega
KPHS_52130	0.17	0.08	1.07	0.35	1.88	up	1.13E-04	N-acetylmuramic acid phosphotransfer permease
KPHS_p200470	1.85	1.66	0.00	0.65	1.91	down	1.29E-02	transposase IS26
KPHS_p200640	3.78	2.38	9.88	9.65	1.91	up	3.78E-18	beta-lactamase
KPHS_p201390	4.88	4.70	0.53	1.57	2.04	down	1.67E-07	hypothetical protein
KPHS_p300540	42.81	32.76	145.06	138.31	2.05	up	0.00E+00	beta-lactamase
KPHS_p300840	42.81	32.76	145.06	138.31	2.10	up	0.00E+00	beta-lactamase
KPHS_p300860	9.07	7.59	29.28	28.42	2.24	up	1.26E-54	streptomycin 3'-kinase
KPHS_p300870	14.53	11.92	42.54	46.66	2.36	up	2.81E-79	hypothetical protein
KPHS_p300880	5.76	3.17	17.93	14.96	2.54	up	1.86E-33	Dihydropteroate synthase

Figure S1 The scheme of screening potential antibacterial compounds.

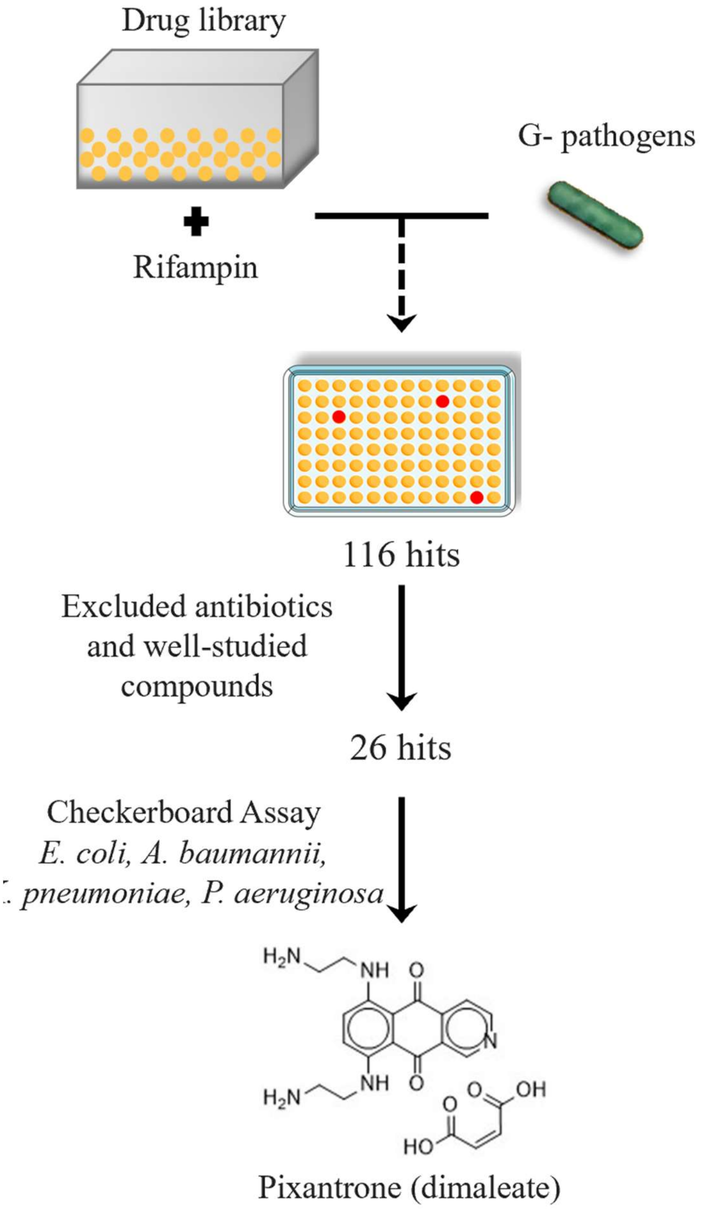

Figure S2 Chemical structure formulas of 26 selected compounds.

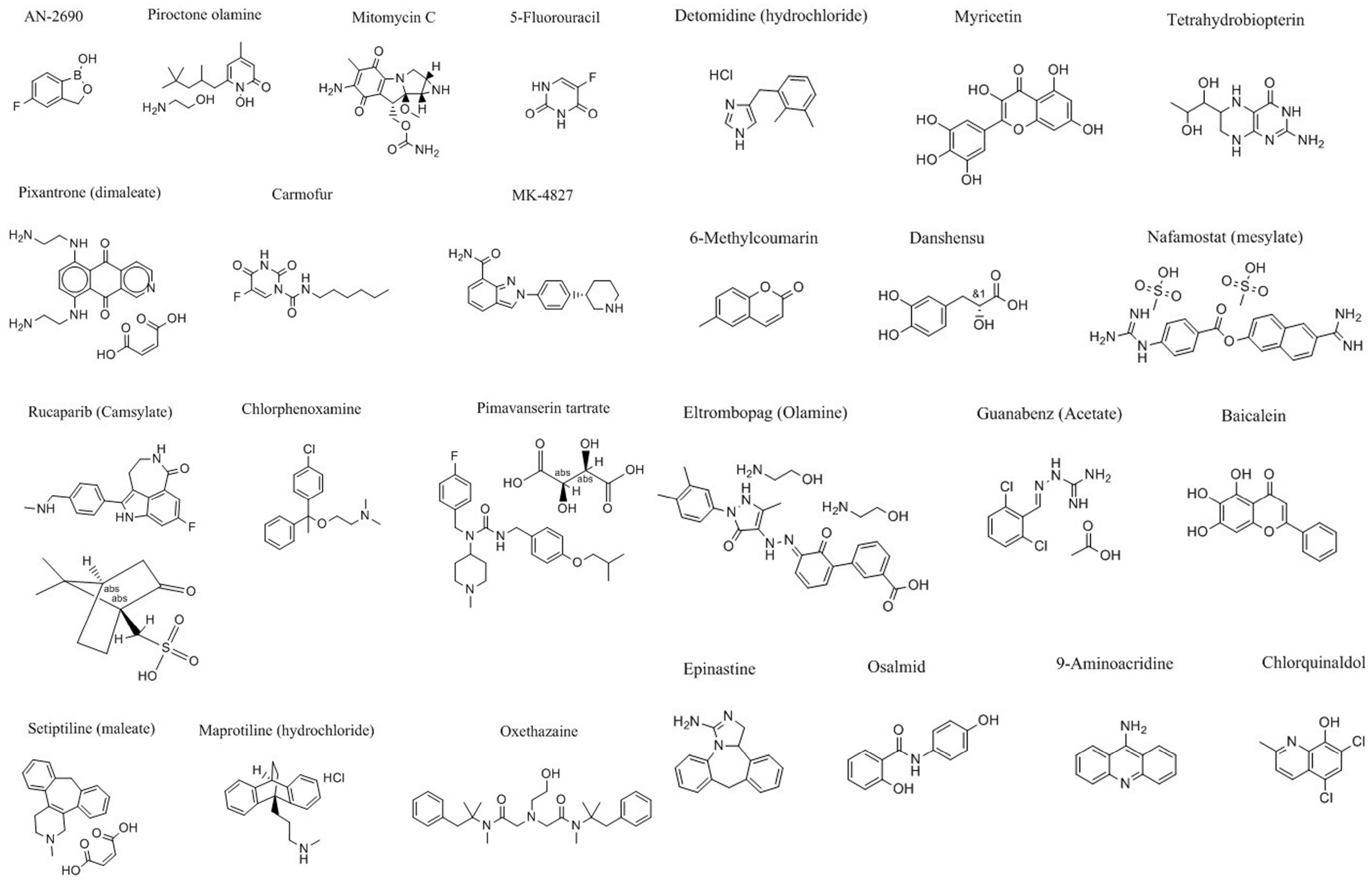

Figure S3 Checkerboard broth microdilution assays between PIX and different classes of antibiotics against *E. coli* ATCC 52922. PB, polymyxin B; FOS, fosfomycin; VAN, vancomycin; G, penicillin; CRO, carbapenem; AMP, ampicillin; CLA, clarithromycin; CLI, clindamycin; AZM, azithromycin; IMP, imipenem; TET, tetracycline; LZD, linezolid; GEN, gentamicin.

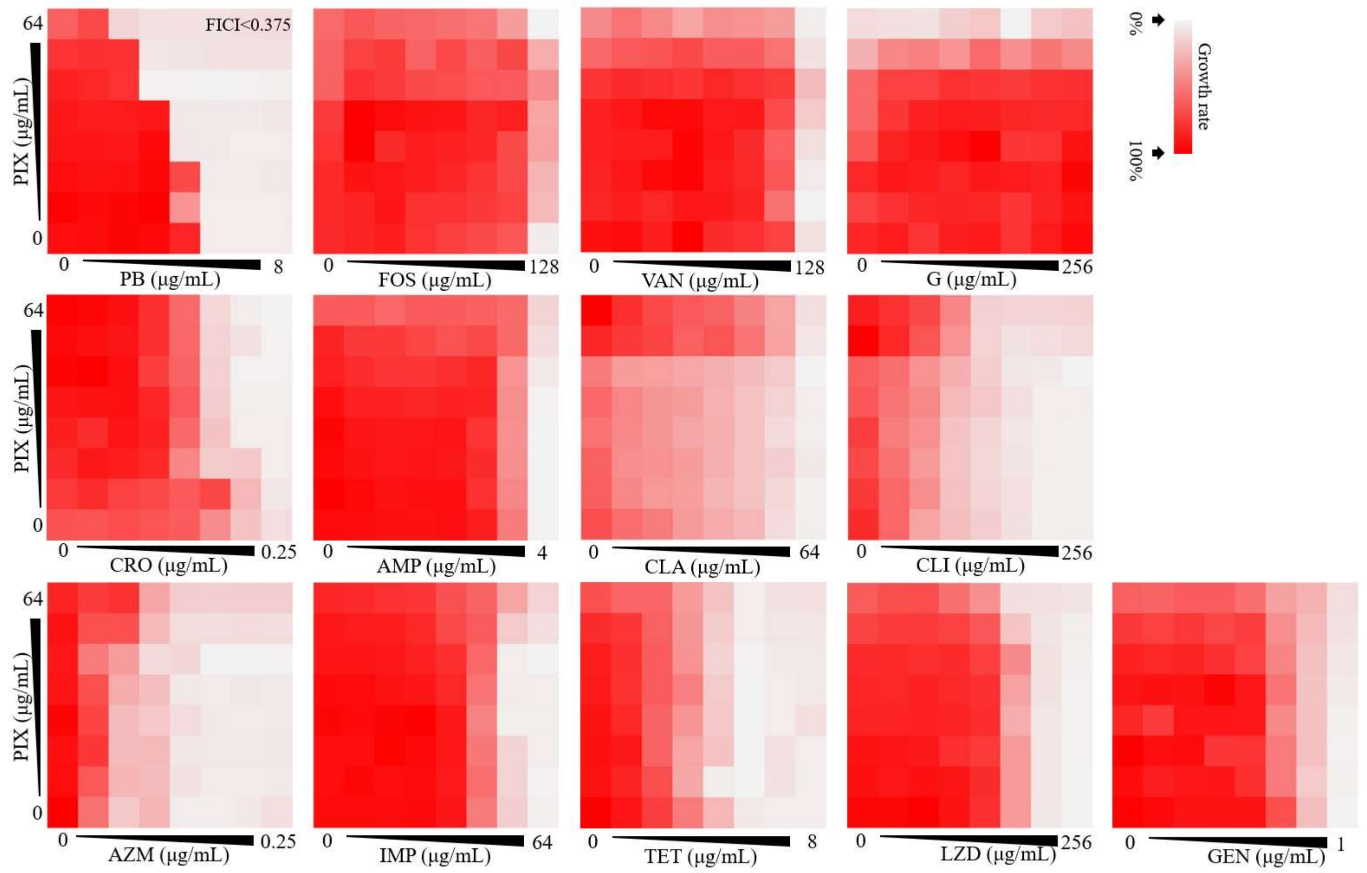

Figure S4 Checkerboard assays of PIX and RFP against representative gram-positive cocci, including *S. aureus* USA300 and *E. faecalis* ATCC 29212.

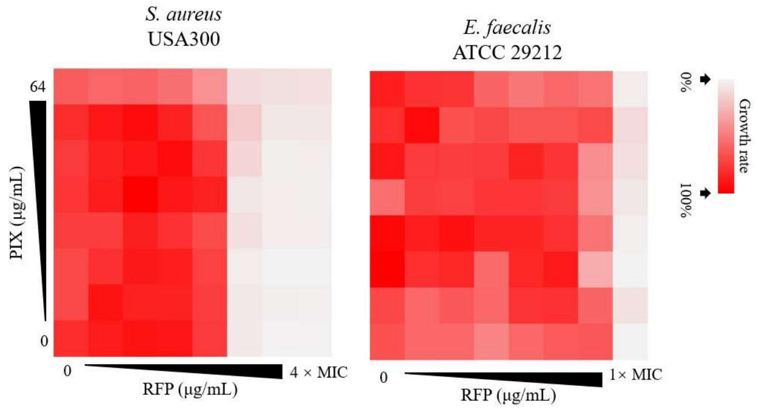

Figure S5 Effect on the bacterial cytoplasmic membrane was observed after PIX treatment. (A) SYTOX Green, (B) PI and (C) DiSC3(5) staining were used to determine the cytoplasmic membrane permeability and membrane potential of four type strains after treatment with various concentrations of PIX. PB, polymyxins B (8 μg/mL); POS, positive control (Melittin. 16 μg/mL). *: p<0.05; **: p<0.01; ***: p<0.001; ****: p<0.0001.

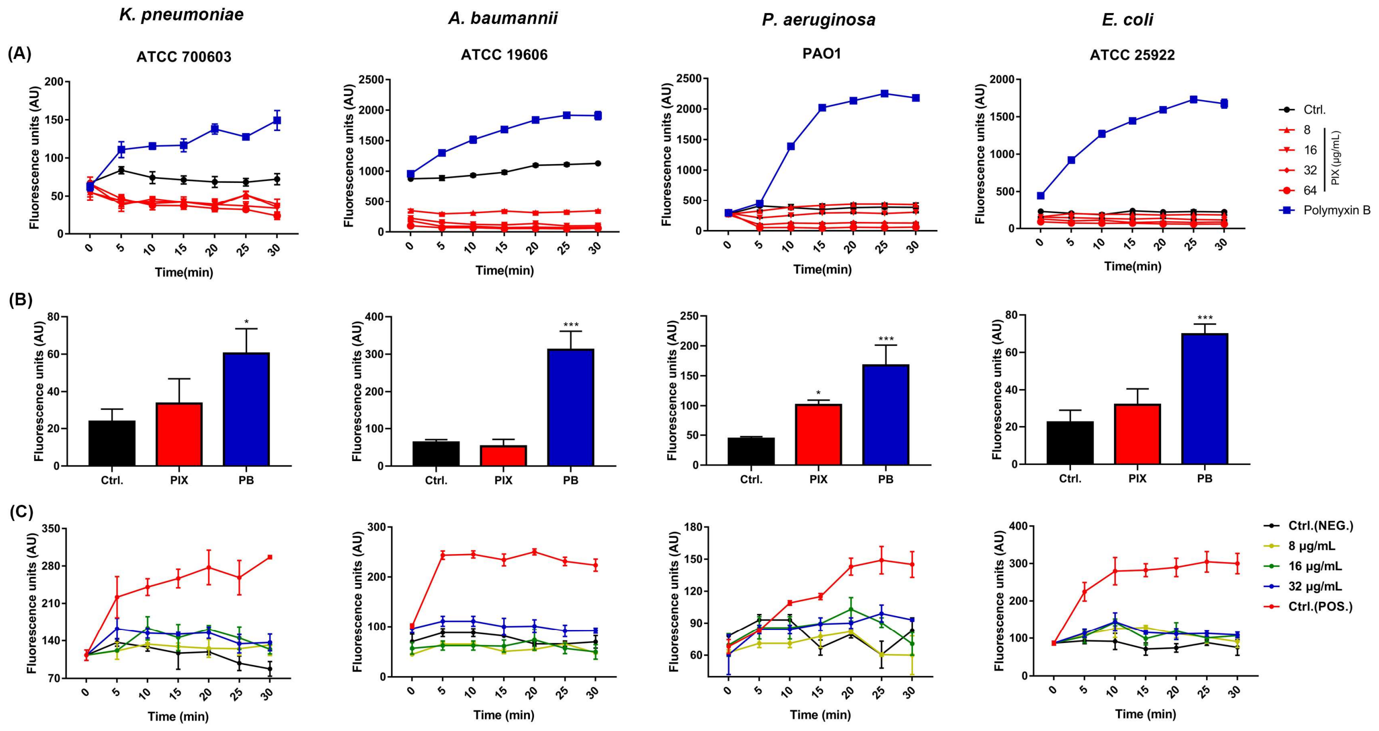

Figure S6 The antimicrobial susceptibility of EDTA and Mg^{2+} against *E. coli* ATCC 25922.

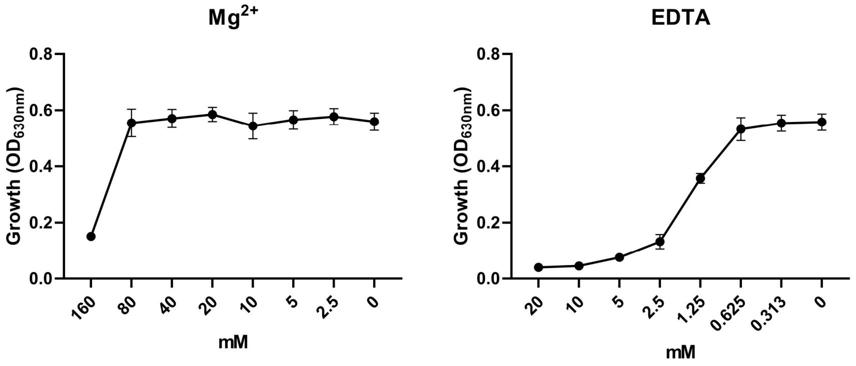

Figure S7 Quantification of surface roughness in AFM. Data are presented as percentages, with the mean value of the control group as 100%.

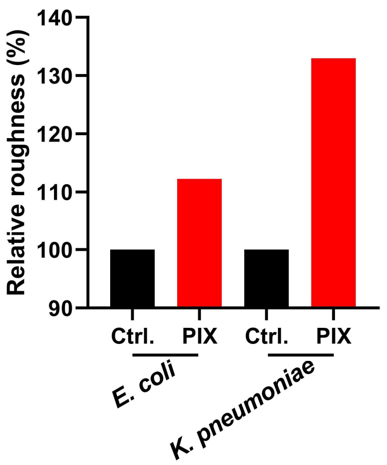

Figure S8 ROS level detection in four XDR gram-nagetive strains, including *K. pneumoniae* KPWANG, *A. baumannii* AB1069, *P. aeruginosa* PA1 and *E. coli* Y9592. PB, polymyxins B (16 μg/mL).

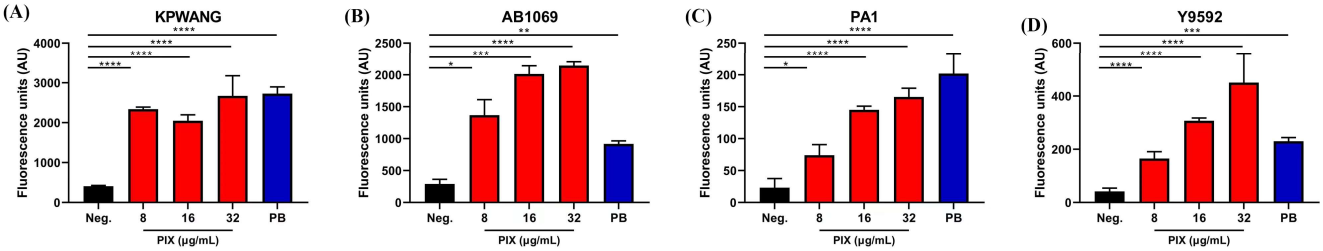

Figure S9 Detection of the intracellular pH by BCECF-AM in gram-nagetive type strains and XDR isolates.

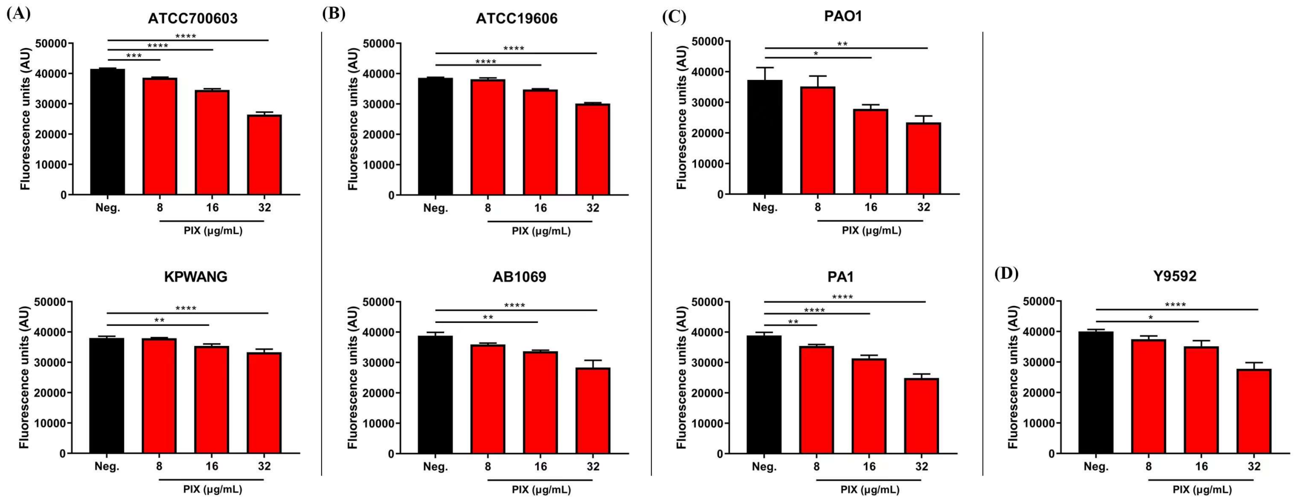

Figure S10 Checkerboard assays between PIX with doxycycline (A) or kanamycin (B) against gram-negative bacteria.

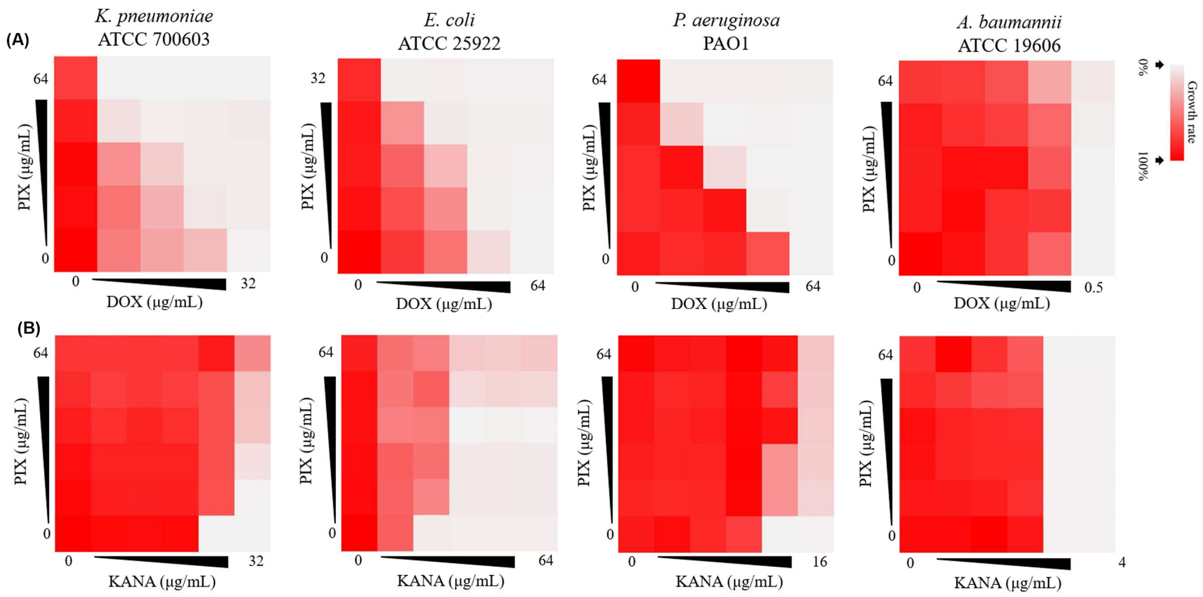

Figure S11 Swimming motility assay in the presence of PIX (16 μg/mL). *: p<0.05; **: p<0.01; ***: p<0.001; ****: p<0.0001.

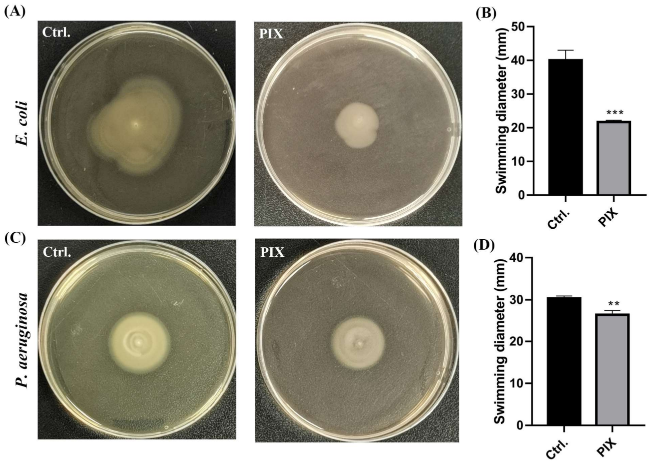

Figure S12 Cytotoxicity determination by PIX. (A) Hemolysis of human RBCs treated with PIX at concentrations up to 256 μg/mL. Triton X-100 (0.1%) and DMSO (1%) were used as positive and negative controls, respectively. CCK-8 assays were used to detect the cytotoxicity of PIX in (B) BEL-7404, (C) RAW264.7, (D) HMC3, (E) HK-2, (F) U251 and (G) 786-O cells. Effects of (H) cisapride (positive control) and (I) PIX on hERG channels by the manual patch-clamp method.

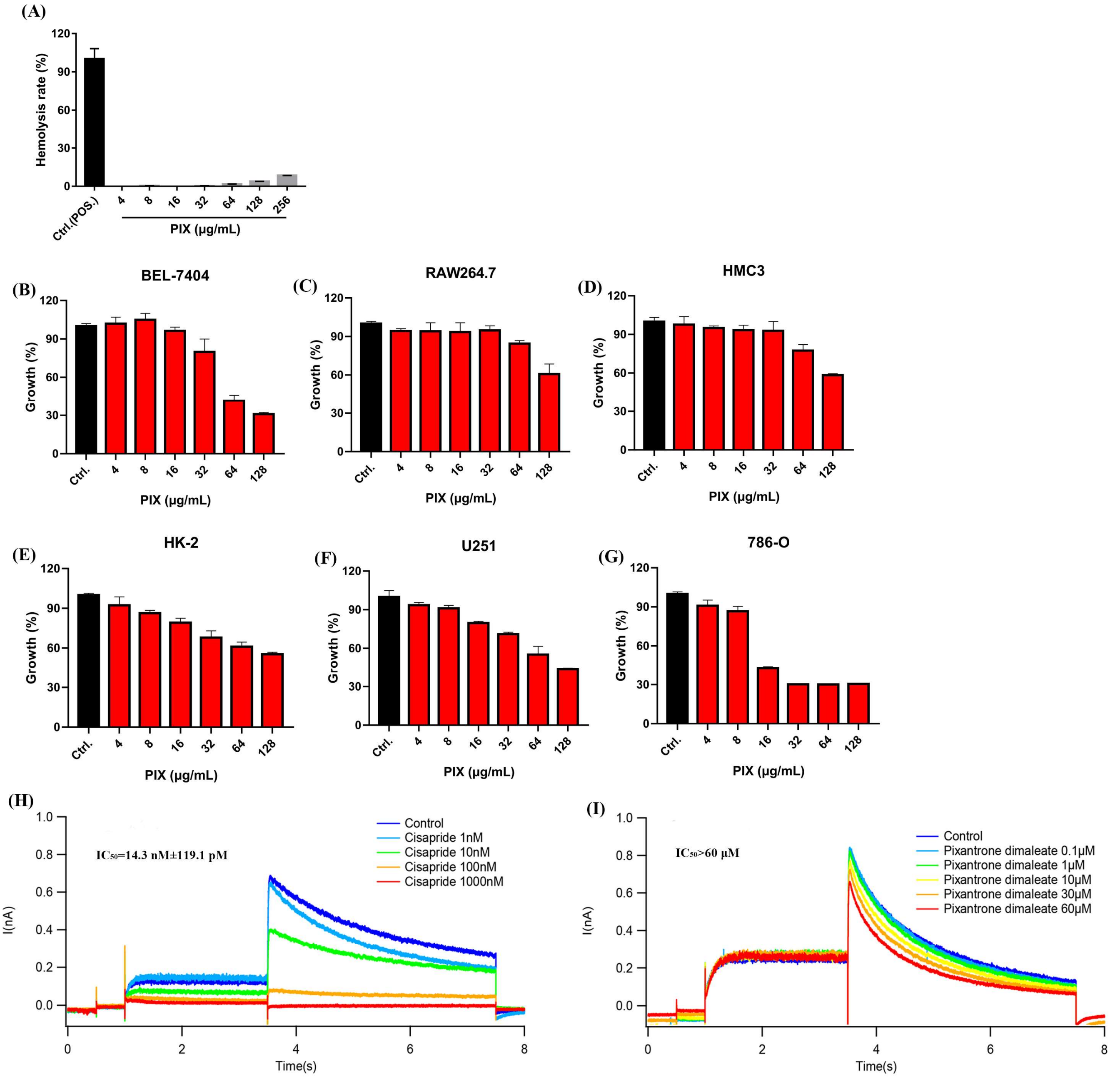

Figure S13 Detection of the apoptosis-inducing ability of PIX (32-128 μg/mL) in RAW264.7 cells. Cultures were stained with Annexin V–FITC and PI and imaged by CLSM. BF, bright field. Scale bar: 100 μm.

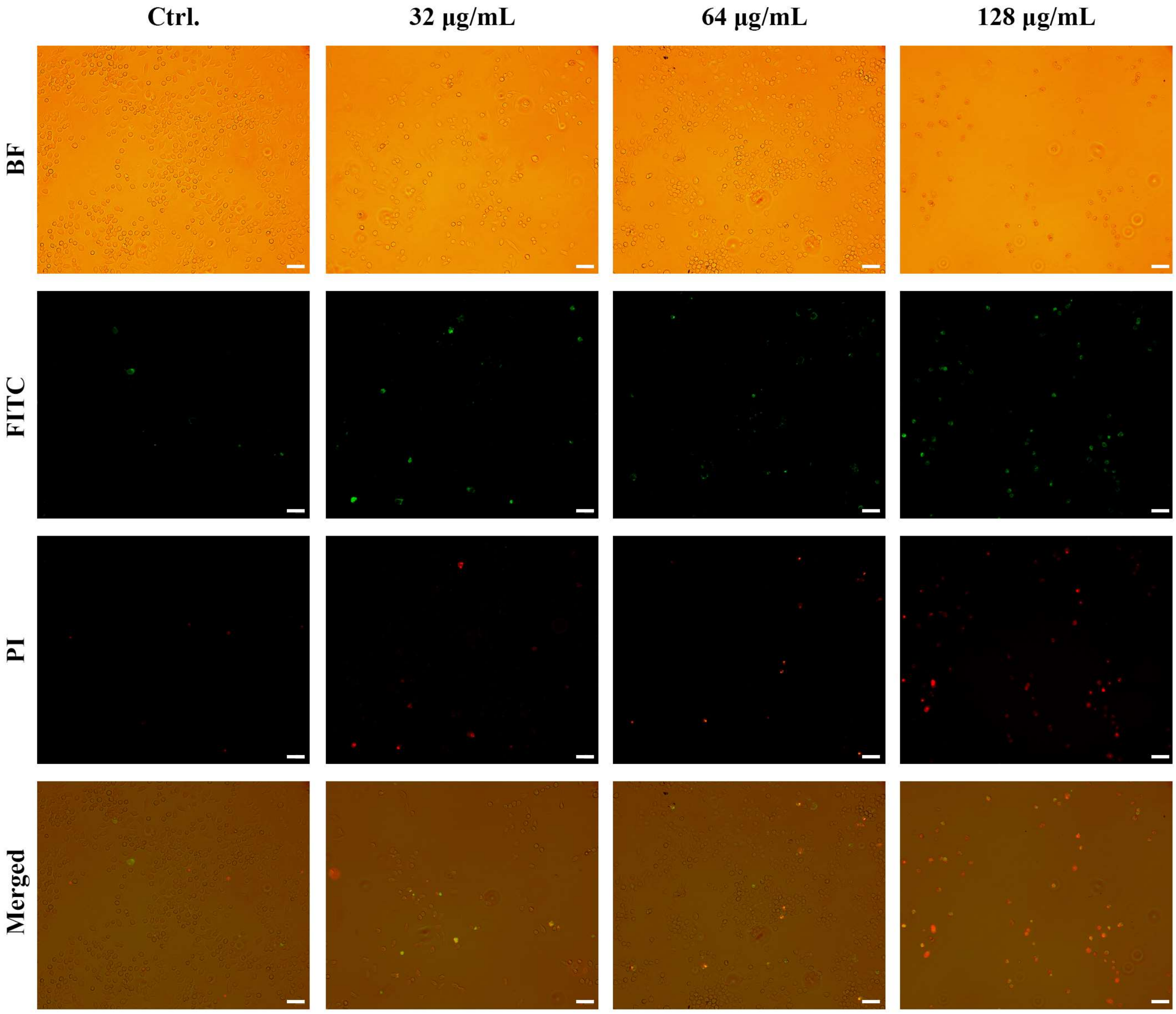

Figure S14 *In vitro* safety. (A-D) After treatment with PIX (16-32 μg/mL), LO2 or HepG2 cells were stained with FITC-conjugated Annexin V and PI, and apoptosis was detected by cytometry. (E-F) LO2 or HepG2 cells treated with PIX (16-32 μg/mL) were stained with DCFH-DA and analyzed by flow cytometry. (I-J) The expression of actin and tubulin in LO2 cells treated with PIX was detected by WB.

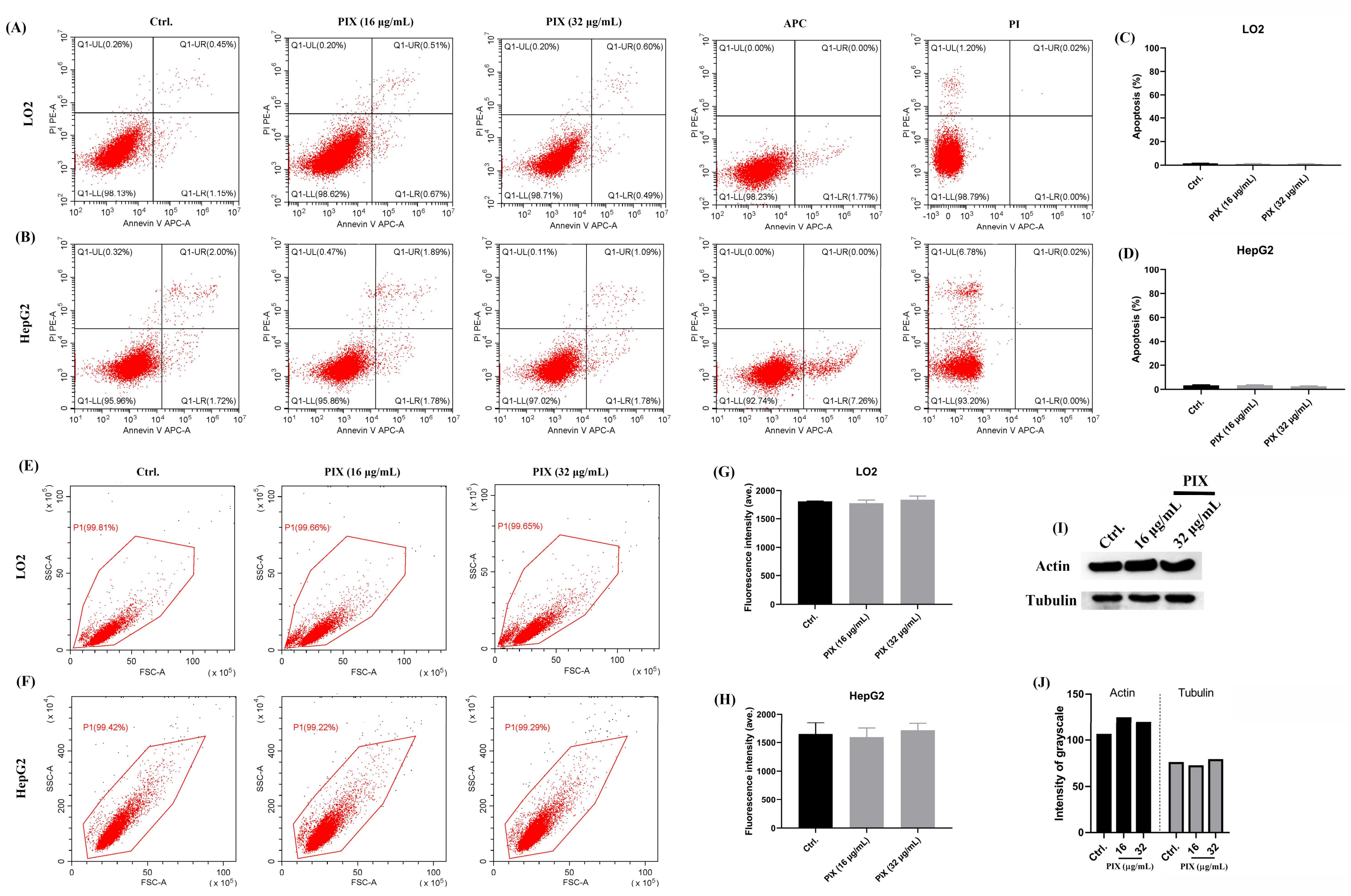

Figure S15 Using DCFH-DA to detect ROS generation following treatment with PIX (32 μg/mL) in mammalian cells, including (A) HepG2 and (B) LO2 cell lines. Neg, negative control (0.1% DMSO); Pos, positive control. Scale bar: 100 μm.

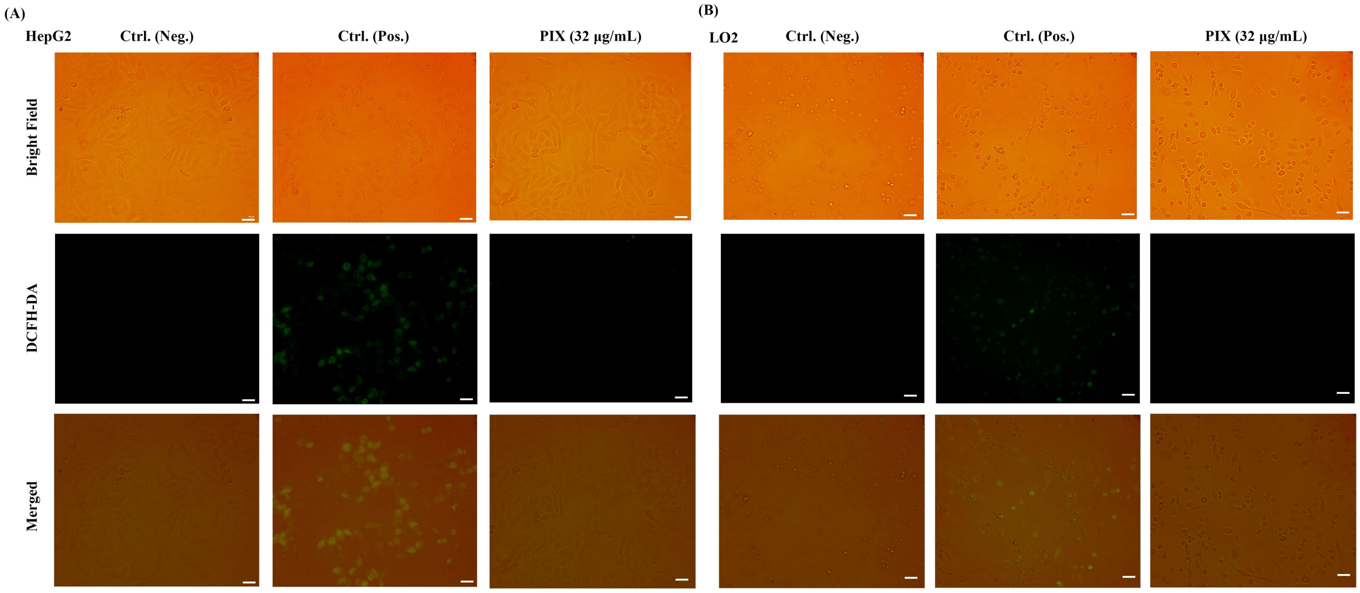

Figure S16 *In vivo* safety analysis. (A) Survival rates of ICR mice (n=5) administered a single dose of PIX (0, 60, 100, 200 or 400 mg/kg, i.p.). Alterations in the (B) hematological parameters, (C) myocardial, (D) kidney and (E) liver biomarkers in mice following administration of 0.1% DMSO (untreated), RFP (20 mg/kg) or RFP + PIX (20+30 mg/kg), respectively. Data are presented as percentages, with the mean value of the untreated group as 100%. WBC, white blood cell counting; RBC, red blood cell counting; HGB, hemoglobin quantification; PLT, platelet counting; N%, quantification of neutrophil percentage; CK, serum creatine kinase; Cr, serum creatinine; BUN, serum urea nitrogen; UA, serum uric acid; ALT, alanine aminotransferase; AST, aspartate aminotransferase; ALB, albumin. (F) H&E staining analysis of the heart, liver, spleen, lung, and kidney after treatment with PIX alone or in combination with RFP. Scale bar: 200 μm.

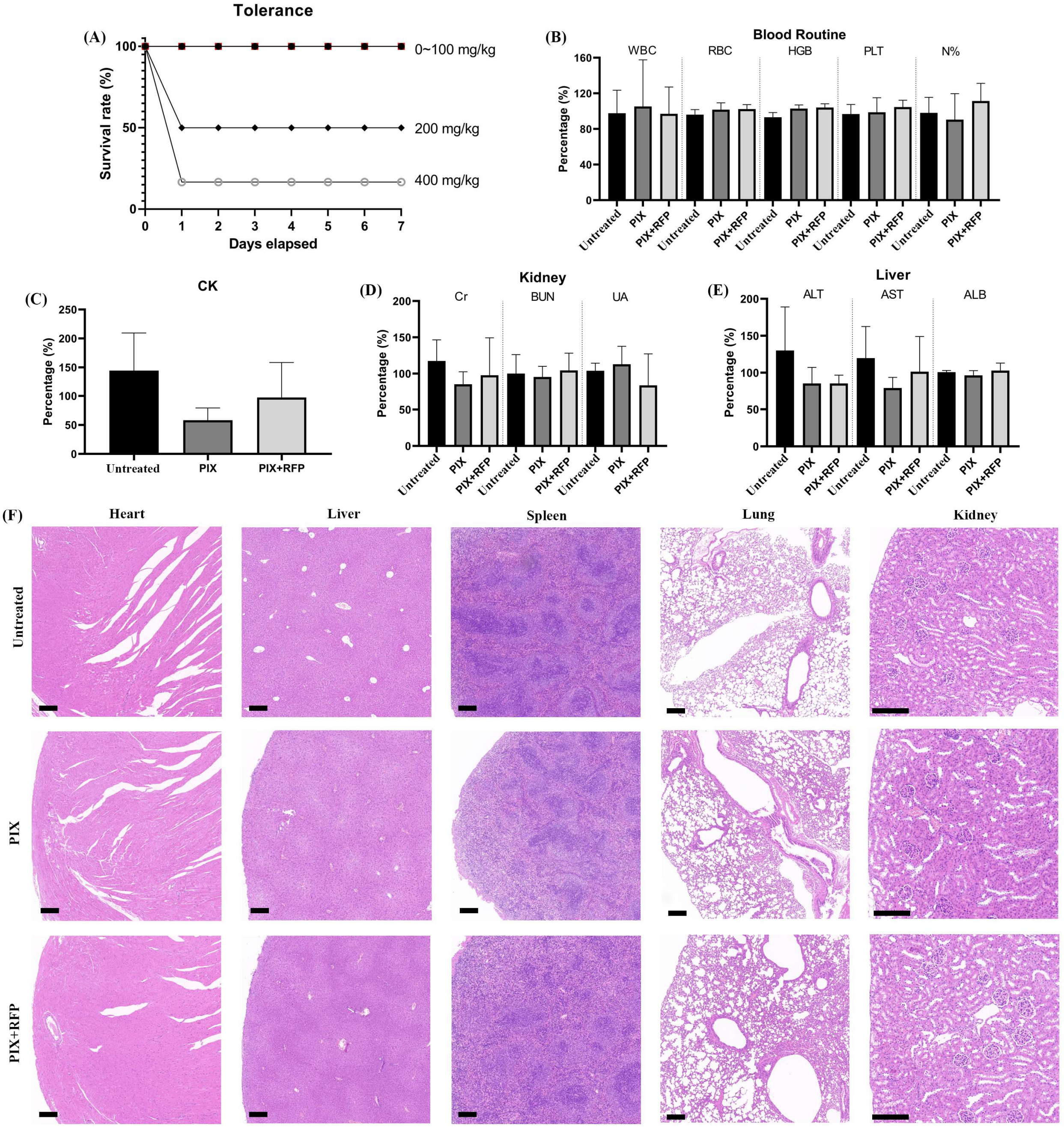

Supplement: Supplemental file 1 — Tables S1 to S3 and Fig. S1 to S16. Download spectrum.02114-22-s0001.pdf, PDF file, 6.1 MB [file spectrum.02114-22-s0001.pdf]
